# A New Family of Predicted Krüppel-Like Factor Genes and Pseudogenes in Placental Mammals

**DOI:** 10.1371/journal.pone.0081109

**Published:** 2013-11-07

**Authors:** Jimin Pei, Nick V. Grishin

**Affiliations:** 1 Howard Hughes Medical Institute, University of Texas Southwestern Medical Center, Dallas, Texas, United States of America; 2 Department of Biophysics and Department of Biochemistry, University of Texas Southwestern Medical Center, Dallas, Texas, United States of America; Nanjing Medical University, China

## Abstract

Krüppel-like factors (KLF) and specificity proteins (SP) constitute a family of zinc-finger-containing transcription factors that play important roles in a wide range of processes including differentiation and development of various tissues. The human genome possesses 17 KLF genes (*KLF1*–*KLF17*) and nine SP genes (*SP1*–*SP9*) with diverse functions. We used sequence similarity searches and gene synteny analysis to identify a new putative KLF gene/pseudogene named *KLF18* that is present in most of the placental mammals with sequenced genomes. *KLF18* is a chromosomal neighbor of the *KLF17* gene and is likely a product of its duplication. Phylogenetic analyses revealed that mammalian predicted KLF18 proteins and KLF17 proteins experienced elevated rates of evolution and are grouped with KLF1/KLF2/KLF4 and non-mammalian KLF17. Predicted KLF18 proteins maintain conserved features in the zinc fingers of the SP/KLF family, while possessing repeats of a unique sequence motif in their N-terminal regions. No expression data have been reported for *KLF18*, suggesting that it either has highly restricted expression patterns and specialized functions, or could have become a pseudogene in extant placental mammals. Besides *KLF18* genes/pseudogenes, we identified several *KLF18*-like genes such as *Zfp352*, *Zfp352*-like, and *Zfp353* in the genomes of mouse and rat. These *KLF18*-like genes do not possess introns inside their coding regions, and gene expression data indicate that some of them may function in early embryonic development. They represent further expansions of KLF members in the murine lineage, most likely resulted from several events of retrotransposition and local gene duplication starting from an ancient spliced mRNA of *KLF18*.

## Introduction

Krüppel-like factors (KLF) and specificity proteins (SP) are an important family of transcription factors (SP/KLF family) under extensive research [1–3]. They possess three DNA-binding C2H2-type zinc finger domains, each of which contains two conserved cysteines and two conserved histidines for zinc binding. The three zinc finger domains and the linkers between them are well conserved in the SP/KLF family, with a cysteine-histidine pattern of “CX_4_CX_12_HX_3_HX_7_CX_4_CX_12_HX_3_HX_7_CX_2_CX_12_HX_3_H” (X_*n*_: separation of *n* residues). The separations between the first, second, and third cysteine pairs are four residues, four residues, and two residues, respectively. Such a pattern together with the number of C2H2 domains (three) appears to be a unique feature of SP/KLF members in mammalian genomes compared to the patterns of other known C2H2-domain-containing proteins (based on an analysis of human and mouse C2H2-domain-containing proteins from the SysZNF database [4]). For example, the EGR2 protein has three zinc fingers but exhibits a different pattern of residue separations between cysteine pairs (4, 2, 2 residue separations compared to 4, 4, 2 residue separations in SP/KLF members). Wilms’ tumor protein possesses four C2H2 domains: three of them sharing the same pattern as the SP/KLF proteins (4, 4, 2 residue separations between cysteine pairs) and a C-terminal fourth C2H2 domain with a four-residue separation between the cysteines. The SP/KLF family proteins mainly recognize and bind GC-rich regions such as GC boxes and GT boxes (CACCC boxes). The structure of KLF4 zinc finger domains in complex with DNA [5] revealed conserved residues responsible for specific DNA interactions. Among them are three invariant arginines that use their guanidinium groups to form critical hydrogen bonds with three guanine bases and contribute the most to the DNA-binding specificity of KLFs. In contrast to the high sequence conservation in zinc fingers, the N-terminal regions of KLFs exhibit great sequence variation [3,6]. These regions contain short sequence motifs that mediate the interactions between KLFs and other proteins such as transcription coactivators and corepressors.

The SP/KLF family proteins regulate a diverse array of cellular processes in development, differentiation, and cell death. Some human SP/KLF members have been associated with various diseases [6]. A total of 17 KLF genes (*KLF1*-*KLF17*) and nine SP genes (*SP1*–*SP9*) are currently annotated in the human genome. Compared to KLF proteins, SPs are characterized by a unique cysteine-rich motif (“CXCPXC”, buttonhead box) in the region N-terminal to the zinc fingers. Several phylogenetic analyses of the SP/KLF family proteins [2,7] suggest that SPs form a monophyletic group and are more closely related to a subgroup of KLF proteins (e.g., KLF9/KLF13/KLF14/KLF16) than to the other KLF proteins. Members of the SP/KLF family differ in their tissue expression patterns and their functions. Some KLF genes, such as *KLF3*, *KLF9* and *KLF10*, exhibit broad expression patterns, while other members are expressed in restricted tissues. For example, human *KLF1* (also named erythroid KLF, or *EKLF*) is mostly expressed in erythroid cells and regulates their differentiation.

Of the 17 human KLF genes, three members (*KLF1*, *KLF14*, and *KLF16*) appear to be mammalian-specific. The other human KLF genes have orthologs in other vertebrates such as chicken, frog, and teleost fish. SP/KLF members have also been identified in metazoans outside vertebrates, albeit the number of SP/KLF genes in these species is smaller [7,8]. Two rounds of whole-genome duplications in the ancestor of vertebrates [9] may partially explain the increased number of SP/KLF genes in vertebrates. A more recent whole-genome duplication event in the ancestor of teleost fish could have resulted in highly similar KLF pairs in *Danio rerio*, such as KLF5a/KLF5b and KLF15a/KLF15b.

The most recent mammalian gene assigned to the SP/KLF family, *KLF17* [10], was first discovered as a germ cell-specific gene encoding zinc finger protein 393 (Zfp393) in mouse [11]. Human and mouse KLF17 proteins exhibit less sequence similarity compared to other orthologous KLF protein pairs, suggesting that KLF17 has undergone rapid evolution in the mammalian lineage [10]. This relatively high sequence divergence of KLF17s compared to known KLF proteins has delayed the inference of KLF17 as a KLF member [10]. Based on gene synteny, *KLF17* was also proposed to exist in non-mammalian species [12]. Specifically, mammalian, chicken, and frog *KLF17* genes are sandwiched by the *SLC6A9* gene and the *DMAP* gene [12]. Likewise, a fish KLF17 ortholog can be inferred based on the syntenic similarity of a fish gene [13] (NCBI Gene ID: 65238, previously proposed to be *KLF4* [14] and later annotated as *Klf4b* in the NCBI gene database) to *KLF17* genes in other vertebrates.

We combined sequence similarity searches, multiple sequence alignment, phylogenetic reconstruction, and gene synteny analysis for computational identification of new KLF genes/pseudogenes in mammals. We predicted a novel KLF gene or pseudogene, named *KLF18*, in most of placental mammals with sequenced genomes including human and mouse. Mammalian *KLF18* and *KLF17* are chromosomal neighbors, and their inferred protein products form a monophyletic group to the exclusion of other known KLF proteins, suggesting that *KLF18* resulted from a local gene duplication of *KLF17*. We propose that *KLF18* retrotransposition and local gene duplication resulted in further expansion of KLF members in the murine genomes of mouse and rat, giving rise to the highly diversified *Zfp352*, *Zfp352l*, and *Zfp353* genes [15]. 

## Materials and Methods

### Mammalian genome selection

To study the distribution of predicted *KLF18* genes/pseudogenes, we examined 44 mammalian genomes available in the UCSC genome browser [16] as of December 2012. For three species, sheep, hedgehog, and tenrec, we used their latest genome assemblies from NCBI with higher coverage of sequencing than their UCSC versions. In addition, we also analyzed NCBI genome assemblies of three recently sequenced mammalian species from the Afrotheria superorder, which like the Xenarthra superorder, is underrepresented compared to the other two placental mammalian superorders (Euarchontoglires and Laurasiatheria). The total number of mammalian genomes analyzed is 47, consisting of 43 placental mammals and four non-placental mammals (Table S1).

### Protein similarity searches and phylogenetic analyses of predicted KLF proteins

BLAST [17] was used to search for close homologs of KLF proteins starting with known human KLF proteins against the nr database in NCBI (e-value cutoff: 1e-10). Multiple sequence alignment of KLF proteins was made by MAFFT [18] (options: --localpair --maxiterate 1000). For phylogenetic analyses, we selected a set of SP/KLF proteins consisting of known human KLF proteins (KLF1-KLF17) and SP proteins (SP1–SP9), three non-mammalian KLF17s (from zebrafish, frog and chicken), three predicted KLF18s (from human, mouse and rat), mouse and rat Zfp352 proteins and their close homologs, and the human Wilms’ tumor protein (WT1) as an out-group (WT1 contains four zinc fingers, three of which exhibit the same pattern as SP/KLF members and have a similar set of DNA-binding specificity residues as SP/KLF proteins). The MOLPHY package [19] was used for phylogenetic reconstruction for the zinc finger regions of these proteins. The JTT amino acid substitution model [20] was used in MOLPHY. The local estimates of bootstrap percentages were obtained by the RELL method [21] (-R option in the ProtML program of MOLPHY). For this dataset, we also used MrBayes [22] to run a Bayesian inference of phylogeny using mixed amino acid substitution model with the invgamma (invariant site + gamma distribution of rate variation) option. A total of 300,000 generations were performed, and the first 150,000 generations (50%) were discarded as burn-in. A consensus tree was obtained for the remaining generations with sampling frequency set to one sample per 100 generations. We also applied MOLPHY to a larger dataset of SP/KLF zinc finger regions consisting of known human and zebrafish SP/KLFs, a larger set of predicted KLF18 proteins, and close homologs of mouse and rat Zfp352 proteins.

### Detection of predicted *KLF18* genes/pseudogenes

Translated BLAT [23] was used to search for *KLF18* genes/pseudogenes for UCSC genomes, and TBLASTN [24] was used for the NCBI genomes. Their chromosomal locations were further confirmed by BLAT/TBLASTN searches of *KLF17* and *DMAP1*, two genes neighboring to the *KLF18* locus. For a few species, the pseudogene status of *KLF18* was inferred based on the presence of premature stop condons inside the regions encoding zinc fingers. Pre-calculated gene prediction results available in the UCSC genome browser, mostly by GENSCAN [25], were examined in regions corresponding to the predicted *KLF18* genes. For genomes where such predictions are not available, FGENESH [26] was used to predict *KLF18* genes. TBLASTN was further used to search for missing pieces of zinc finger regions for some predicted *KLF18* genes. The gene prediction results are shown in Table S1, and the predicted KLF18 protein sequences are available in Figure S2.

## Results and Discussion

### *KLF18* is a new predicted KLF gene/pseudogene in most of the placental mammals

**Figure 1:**
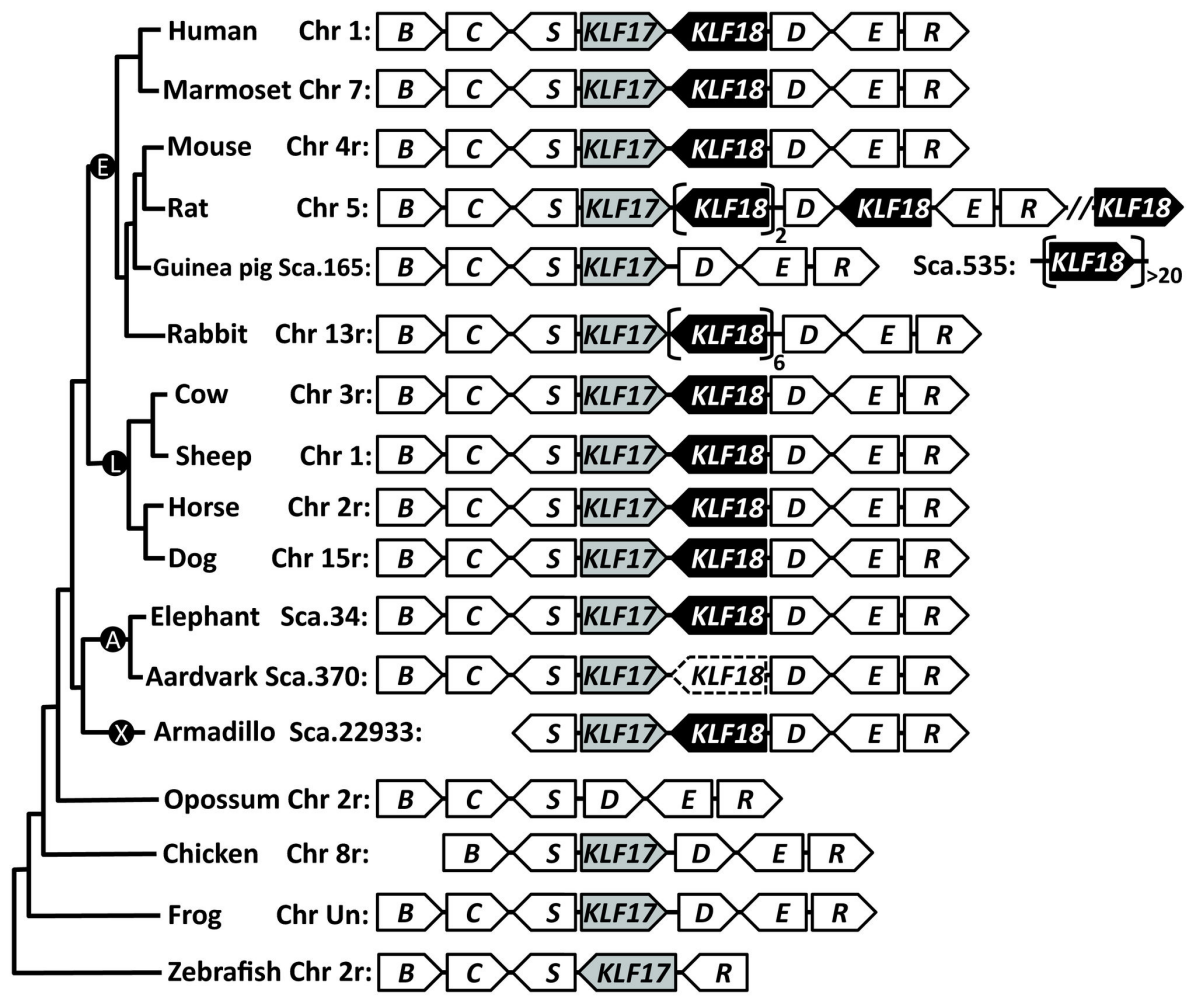
Chromosome localization and gene synteny of *KLF17* and *KLF18* in vertebrate genomes. Chromosome (Chr) or scaffold (Sca.) numbers are shown to the left of the gene order diagrams, with ‘r’ after the chromosome number denoting the reverse strand. In most of the placental mammalian genomes, *KLF17* and *KLF18* are neighbors arranged in a tail-to-tail fashion, and they are sandwiched by three upstream genes (abbreviations: *B*: *B4GALT2*; *C*: *CCDC24*; and *S*: *SLC6A9*) and three downstream genes (abbreviations: *D*: *DMAP1*; *E*: *ERI3*; and *R*: *RNF220*). Such a gene context for *KLF17* is largely preserved in non-mammalian vertebrates including chicken, frog, and zebrafish. Copy number expansions of *KLF18* (the number of expanded genes shown beside the brackets) were observed in rat, guinea pig, and rabbit. The aardvark *KLF18* with pseudogene evidence is shown with dashed outline. The tree on the left shows the relationships of mammals and other vertebrates. Roots of four major groups (superorders) of placental mammals are shown in circles - E: Euarchontoglires; L: Laurasiatheria; A: Afrotheria; and X: Xenarthra.

BLAST sequence similarity searches using zinc finger domains of known human KLF proteins identified several predicted proteins from rabbit annotated as “PREDICTED: mCG120027-like” with e-values ( less than 1e-20) comparable to those of other SP/KLF family proteins. For example, a BLAST search using the human KLF17 protein as the query found a rabbit mCG120027-like protein (Genbank: XP_002715727.1) with an e-value of 5e-25 (score: 118 bits), which is comparable to or better than the e-values of some known KLF proteins (e.g., human KLF8 with an e-value of 1e-24 and human SP4 with an e-value of 4e-24) and better than the e-values of other zinc-finger-containing proteins such as Wilms’ tumor protein (e.g., human Wilms’ tumor protein with an e-value of 6e-19). These predicted rabbit proteins have three C-terminal zinc finger domains with the same cysteine-histidine pattern (“CX_4_CX_12_HX_3_HX_7_CX_4_CX_12_HX_3_HX_7_CX_2_CX_12_HX_3_H”) that is a distinct feature of the SP/KLF family proteins. The names of these rabbit proteins indicate orthology to a predicted mouse protein called mCG120027 (GenBank: EDL30545.1). Potential orthologs of mCG120027 from cow (GenBank: DAA31138.1) and a primate *Otolemur garnettii* (GenBank: XP_003801272.1), both derived from predicted genes, were also among the top BLAST hits of human KLF proteins. Examination of the chromosome locations of these predicted genes revealed that they have conserved gene synteny, as they are all neighbors of *KLF17* genes in corresponding genomes and are oriented in a tail-to-tail fashion compared to *KLF17* genes. Similarity searches against genome sequences of 47 mammals by translated BLAT/BLAST and gene predictions by GENSCAN and FGENESH (see Materials and Methods) identified a predicted mCG120027-like gene or pseudogene downstream of *KLF17* in most of the placental mammals (Figure 1 and Table S1). We name these new predicted genes/pseudogenes (putative orthologs of mouse mCG120027) *KLF18*.

### Is *KLF18* a pseudogene or a protein-coding gene?

*KLF18* was predicted to be a protein-coding gene with zinc finger regions for the majority of the examined placental mammals with sequenced genomes (36 out of 43 genomes, Table S1). *KLF18* pseudogenes were inferred for four genomes of placental mammals (pig, hedgehog, tenrec, and aardvark) based on premature stop codon mutations or deteriorated zinc fingers. KLF18 zinc finger regions were not detected in only three out of the 43 placental mammal genomes. These three genomes (two primates: mouse lemur and bushbaby, and rock hyrax from the Afrotheria group) have low genome sequencing coverage (less than 3 fold) [27]. Verification of *KLF18*’s presence in them may require genome sequences of higher quality. For mouse lemur and rock hyrax, we did find regions with significant similarity to the N-terminal regions (containing a repeated motif, described below) of other predicted KLF18 proteins (Table S1 and Figure S2).

**Figure 2:**
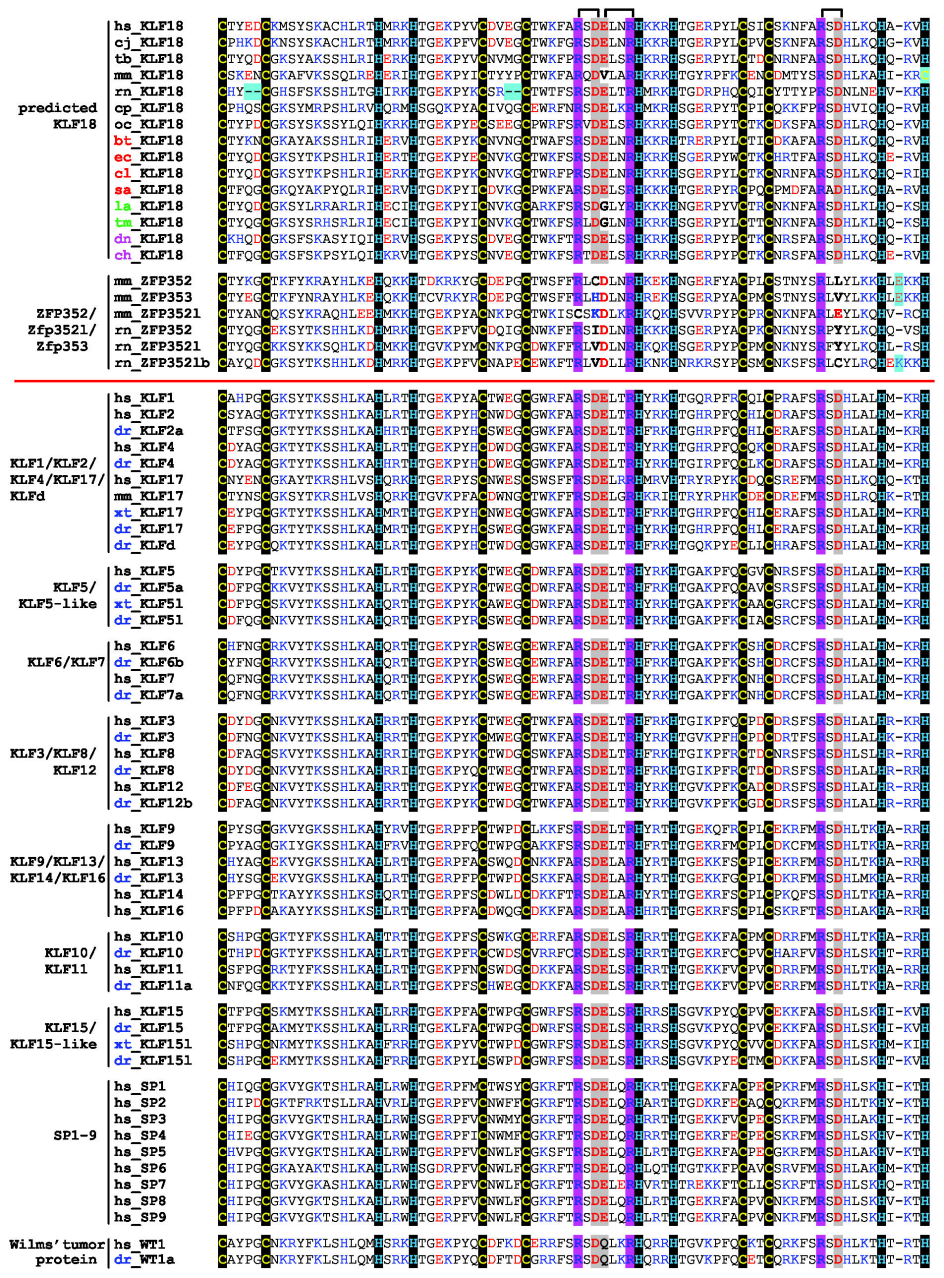
Multiple sequence alignment of three zinc fingers of select KLF proteins and Wilms’ tumor proteins. Two new KLF groups (predicted KLF18 proteins and Zfp352/Zfp352l/Zfp353) are shown above the red line. Known KLF members below the red line are grouped according to frequently well-supported clusters found in separate phylogenetic studies. Conserved cysteines and histidines involved in metal binding are on black background. Three conserved DNA base-interacting arginines are shaded in magenta. Three negatively charged residues interacting with the three arginines are shaded in grey, with connections for interaction pairs shown above the alignment. Negatively charged residues (aspartate and glutamate) and positively charged residues (lysine, arginine, and histidine) are colored red and blue, respectively. Insertion and deletion events are highlighted in cyan. Species name abbreviations are: bt, *Bos taurus* (cow); cf, *Canis familaiaris* (domestic dog); ch, *Choloepus hoffmanni* (two-toed sloth); cj, *Callithrix jacchus* (common marmoset); cp, *Cavia porcellus* (guinea pig); dn, *Dasypus novemicinctus* (nine-banded armadillo); dr, *Danio rerio* (zebrafish); ec, *Equus caballus* (horse); hs, *Homo sapiens* (human); la, *Loxodonta Africana* (African Savannah elephant); mm, *Mus musculus* (mouse); oc, *Oryctolagus cuniculus* (rabbit); rn, *Rattus norvegicus* (rat); sa, *Sorex araneus* (common shrew); tb, *Tupaia belangeri* (tree shrew); tm, *Trichechus manatus latirostris* (the Floirida manatee); xt, *Xenopus tropicalis* (Western clawed frog). Species names are colored as follows - black: Euarchontoglires; red: Laurasiatheria; green: Afrotheria; magenta: Xenarthra; and blue: non-mammalian vertebrates.

Despite the prevalence of *KLF18* as a predicted protein-coding gene in the majority of the placental mammals analyzed, sequence database searches did not find evidence of gene expression such as cDNA and expressed sequence tags (ESTs) for these predicted *KLF18* genes. Recently, techniques such as RNA-seq [28] and ribosome profiling [29] greatly expanded the data of gene expression. RNA-seq-based data (including ENCODE RNA-seq datasets [30]) supporting *KLF18* expression were not found at the UCSC genome browser [16]. We also searched the NCBI Sequence Read Archive (SRA) for potential transcripts of human *KLF18* and only found a few spurious hits. The lack of expression data suggests that some or all of these predicted *KLF18* genes may not be expressed and may have become pseudogenes. Pseudogene evidence was available for a couple of genomes such as hedgehog and aardvark, as premature stop codons were detected inside the regions corresponding to the C2H2 zinc fingers. However, for the majority of the placental mammal genomes examined, *KLF18* was predicted to be a protein-coding gene by GENSCAN or FGENESH, and their predicted coding regions lack deterioration signals commonly found in pseudogenes such as frame shifts and premature stop codons. Moreover, the predicted KLF18 proteins exhibit conserved features in the zinc finger regions as compared to known KLF proteins (Figure 2 and see Figure S1 for the alignment of zinc fingers of all predicted KLF18 proteins derived from analyzed genomes). In particular, the zinc-binding cysteines and histidines are mostly preserved. One exception is the last zinc-binding position in the mouse KLF18 (predicted protein mCG120027) (Figure 2), where the histidine is replaced by a cysteine residue. In a general C2H2 zinc finger consensus sequence, both histidine and cysteine are allowed in such a position, and thus this change may not affect the zinc-binding potential of mCG120027 if it is translated from the mouse predicted *KLF18* gene.

Besides metal-binding residues, the other parts of the zinc finger domains of the newly predicted KLF18 proteins are also well conserved compared to known KLFs (Figure 2 and Figure S1). Most interestingly, three arginine residues contributing most to the specific interactions with DNA base pairs are conserved in predicted KLF18 proteins, like other SP/KLF members (Figure 2 and Figure S1). These arginines (two in the second zinc finger and one in the third zinc finger, on magenta background in Figure 2) use their side-chain guanidinium groups to make double hydrogen bond interactions with three guanine bases in the consensus GC box/GA box motif (GGCG or GGTG) [5]. Three negatively charge residues (two aspartic acids and one glutamic acid) that help orienting the guanidinium groups of these arginines are also largely preserved in predicted KLF18 proteins (Figure 2 and Figure S1). Therefore, it is likely that predicted KLF18 proteins, if translated, are capable of DNA-binding and recognition of DNA motifs such as GC box and GT box like known KLF proteins.

The coding potential of the predicted exon regions encoding the three zinc fingers of KLF18 was probed by the program PhyloCSF [31] for ten species (human, mouse, rat, guinea pig, rabbit, cow, horse, dog, elephant, and armadillo). PhyloCSF aims to distinguish protein coding regions from non-coding regions based on codon substitution frequencies and does not rely on information of similarity to other proteins. PhyloCSF gave a positive score of 305.9 decibans (a score of *N* decibans corresponds to a difference of 10^0.1^*^N^ fold), suggesting that the protein coding model is ~10^30^ times more likely than the non-coding model. Such a score supports the hypothesis that *KLF18* or at least its ancestral form is a protein coding gene. If *KLF18* is still active in extant mammals, the lack of expression data for *KLF18* suggests very low expression levels or tightly controlled spatial or temporal expression patterns.

### A unique repeated motif in predicted KLF18 proteins

**Figure 3:**
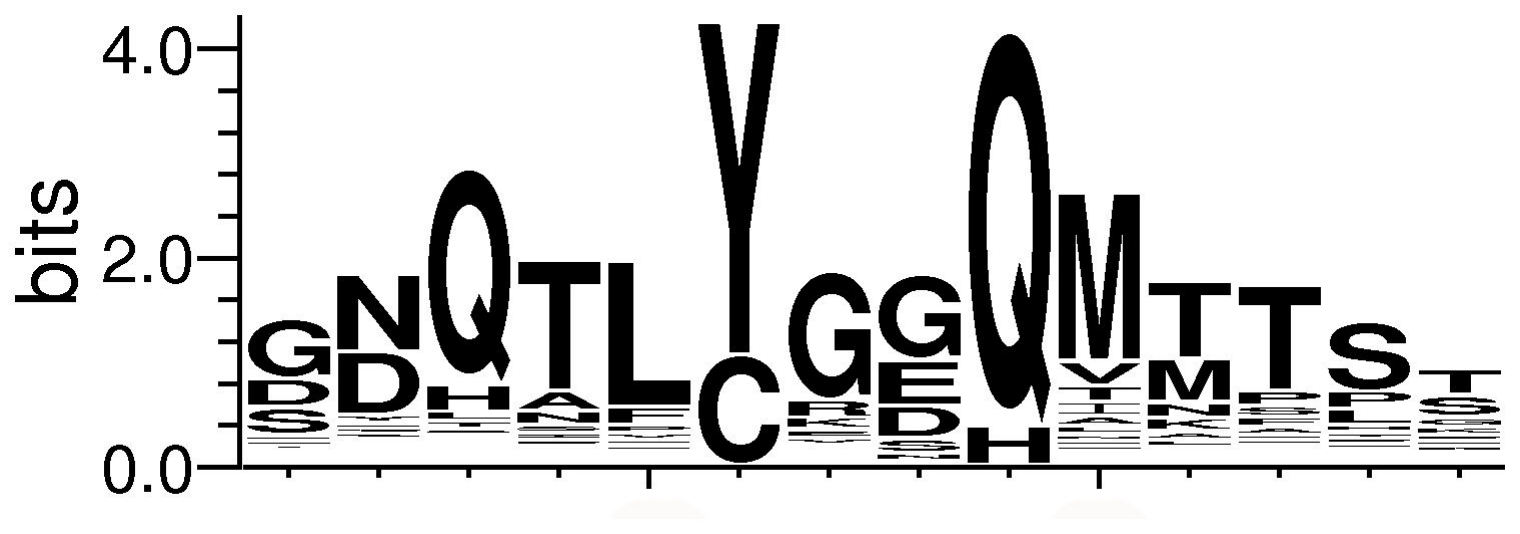
Sequence logo of the repeated segments in the N-terminal regions of predicted KLF18 proteins. Four-residue sequence segments matching the pattern “[YC]x[sE][QH]” are extracted from predicted KLF18 proteins. These segments were extended by five residues both N-terminally and C-terminally to obtain segments of 14 residues. Sequence logo was generated for the expanded segments by the program WebLogo [44].

KLF proteins possess N-terminal regions that are highly variable compared to zinc finger regions [3,6]. Closely related KLFs often share certain short sequence motifs for protein-protein interactions inside these regions. For example, KLF3, KLF8, and KLF12 contain a CtBP-binding site with a sequence consensus of “PXDLS” [3,6,32,33], while another closely related group of KLFs (KLF9, KLF10, KLF11, KLF13, KLF14, and KLF16) contain a Sin3A-binding motif that adopts an alpha-helical structure [6,34–36]. Like other KLFs, predicted KLF18 proteins typically possess a long N-terminal region (most of them larger than 300 amino acids, Figure S2). These regions share little sequence similarity to N-terminal regions of known SP/KLF family members. One interesting feature of such regions in predicted KLF18 proteins is the presence of a unique repeated motif exhibiting the pattern of “[YC]x[sE][QH]” (x: any amino acid, s: a small residue such as Gly, Ala, Ser, Thr, Asp, Asn and Pro, Figure S2). For example, the human and mouse predicted KLF18 proteins have 50 and 14 copies of such repeats, respectively (Figure S2). The first position of this four-residue motif has a preference for tyrosine (Y) with less frequent occurrence of cysteine (C), while the last position of this motif is mostly glutamine (Q). Residue preferences were also observed in positions before and after the motif. For example, the three positions before the conserved tyrosine are most frequently occupied by Q, T, and L, respectively (see sequence logo in Figure 3). Consecutive occurrences of a 14-residue segment, consisting of the [YC]x[sE][QH] motif, five residues before it, and five residues after it, are very common, especially in primate species, e.g. human (Figure S2).

Searches of the human proteome with this motif pattern ([YC]x[sE][QH]) found very few proteins with a high density of this motif (motif density is defined as the number of motifs divided by protein length). Although the cysteine-rich keratins have high concentrations of this motif, their motifs have cysteines in the first position as opposed to mostly tyrosine in predicted KLF18 proteins. Another protein with a high density of this motif is RNA-binding protein 14 (GenBank: NP_006319.1). This protein possesses the “[GS]Y[GS]” repeats often found in proteins from RNA granules [37]. The [YC]x[sE][QH] motifs in this protein overlap with the [GS]Y[GS] motifs, with the residue before the tyrosine being a small residue such as glycine and serine. However, the [YC]x[sE][QH] motif in the predicted KLF18 proteins is different from the [GS]Y[GS] motif since the residue before the first position is often a large hydrophobic residue such as leucine (Figure 3 and Figure S2). As repeated patterns in proteins, such as leucine-rich repeats, heat repeats, and beta helices, are often involved in protein-protein interactions, the repeats in the N-terminal regions of predicted KLF18 proteins may also be responsible for interactions with other proteins, and they may serve to recruit transcription coactivators/corepressors to specific chromosomal locations. However, PSI-BLAST [17] and HHpred [38] searches of several these repeated regions (from human, horse, and elephant) did not yield hits with significant scores to known structures. 

### The origin of *KLF18*

We found *KLF18* in species from all four major groups (superorders) [39] of the placental mammals: Euarchontoglires (including primates such as human and marmoset, rodents such as mouse and rat, and lagomorphs such as rabbit), Laurasiatheria (such as cow, horse, dog, and microbat), Afrotheria (such as elephant and manatee), and Xenarthra (such as armadillo and sloth) (Figure 1, Figure 2, Figure S1, Figure S2, and Table S1). However, sequence similarity searches and gene predictions did not reveal such a gene/pseudogene in non-mammalian vertebrates. *KLF18* was also not found in non-placental mammals (marsupials and monostremes) despite the availability of several genomes of marsupials and the platypus, a monotreme (Table S1). The near-universal presence of *KLF18* in placental mammals but not other genomes suggests that it may have originated in the last common ancestor of extant placental mammals. 

**Figure 4:**
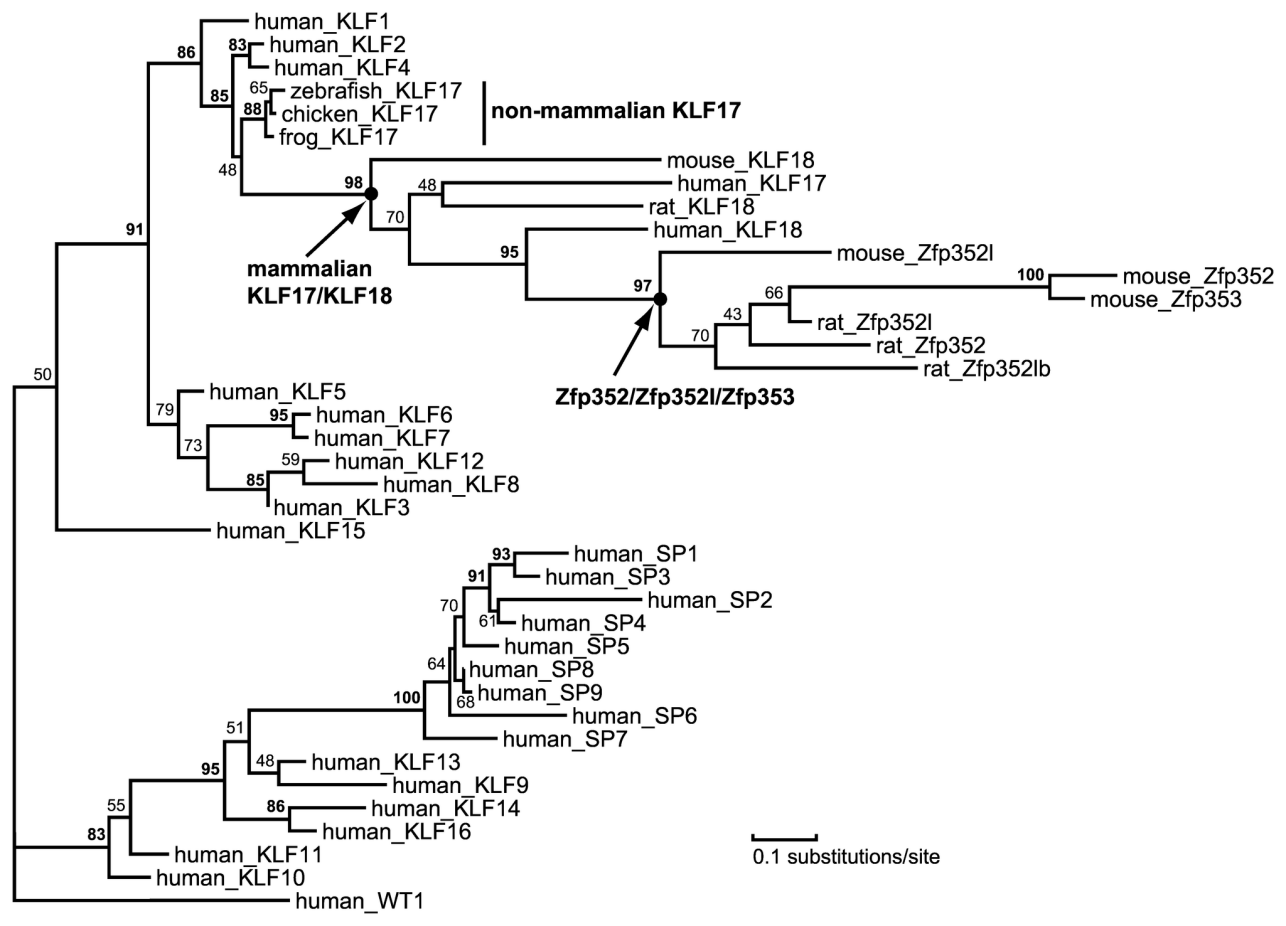
A phylogenetic tree of SP/KLF proteins with the human Wilms’ tumor protein (human_WT1) as an out-group. Branch support values 80 or above are in bold. Each protein node is denoted by the species name followed by the protein name.

A gene structure feature shared by *KLF18*, *KLF17* and most of the other mammalian KLF genes (except *KLF14*) is an intron between the coding regions of first zinc finger and the last two zinc fingers. The intronless *KLF14* gene is believed to be a product of retrotransposition from its close homolog *KLF16* [40]. Presence of an intron in the predicted *KLF18* genes suggests that *KLF18* is not generated by retrotransposition. On the other hand, the closeness of *KLF18* to *KLF17* in chromosomal location (Figure 1) suggests that *KLF18* could have resulted from a local gene duplication of *KLF17*. This scenario of *KLF18* origin is also supported by phylogenetic analyses (Figure 4, Figure S3, and Figure S4, described below), as mammalian KLF17 and KLF18 form a well-supported group to the exclusion of other KLF proteins. 

### Phylogenetic positioning of KLF18 and KLF17

Previous phylogenetic studies consistently identified several well-supported groups of vertebrate KLF proteins, such as KLF1/KLF2/KLF4, KLF3/KLF8/KLF12, KLF6/KLF7, KLF10/KLF11, and KLF9/KLF13/KLF14/KLF16 [2,3,6,7,10,13]. However, the groupings among some of these KLF groups and the positions of some KLF members are not consistently recovered in separate studies. The positioning of mammalian KLF17 is not consistent in several phylogenetic studies [6,7,10,13]. Due to elevated evolutionary rate [10], mammalian KLF17s tend to form long branches in phylogenetic reconstructions. In contrast, a recent phylogenetic study revealed that non-mammalian KLF17 members do not form long branches, and they are grouped with KLF1/KLF2/KLF4 [13]. 

We carried out a maximum likelihood phylogenetic reconstruction (see Materials and Methods) for the zinc finger regions of known human KLF proteins, several vertebrate KLF17 proteins, and some predicted KLF18 proteins and their derivatives (Zfp352, Zfp352l, and Zfp353, described below). In this phylogenetic tree generated by MOLPHY [19], mammalian KLF17s and predicted KLF18 proteins all lie within the group of KLF1/KLF2/KLF4 (Figure 4), like the non-mammalian KLF17s. Mammalian KLF17s, predicted KLF18 proteins, and KLF18 derivatives have much longer branch lengths than other KLF members. Phylogenetic reconstructions by Bayesian analysis using the same dataset and by maximum likelihood on a larger dataset yielded similar results (Figure S3 and Figure S4). 

### Copy number expansions of *KLF18*

*KLF18* was expanded in several genomes of the Glires (rodents and lagomorphs) group, including rat, guinea pig, and rabbit (Figure 1). In each of these genomes, highly similar copies of predicted *KLF18* genes were discovered, suggesting that their copy number expansions have occurred recently and independently. The rat genome has four copies of *KLF18* on chromosome 5, three of which are near *KLF17* (Figure 1). Interestingly, predicted rat KLF18 proteins exhibit a two-residue deletion in each of the first two zinc fingers (Figure 2). Both of such deletions occur between the conserved zinc-binding cysteines (Figure 2). The resulting shorter separations (changed from four residues to two residues) between the conserved cysteines are still allowed in a general zinc finger motif. Such two residue separations are common for C2H2 zinc fingers, e.g., in the third zinc finger of the SP/KLF family members (Figure 2) as well as in members of the SNAIL family [41]. As insertions and deletions rarely occur in zinc fingers of SP/KLF proteins, the deletions in the rat KLF18 are compatible with its elevated evolutionary rate manifested by the long branch length (Figure 4). 

The rabbit genome possesses six highly similar tandem predicted *KLF18* genes near the *KLF17* gene. For guinea pig, we did not identify predicted *KLF18* genes in the assembly scaffold (Scaffold 165) that contains *KLF17* and its surrounding genes (Figure 1). However, on a separate scaffold (Scaffold 635), we found at least 20 tandem repeats of predicted *KLF18* genes. The highly repeated nature of this genome region may have posed challenges for its assembly in the guinea pig genome.

### Expansion of KLF members in the murine genomes by retrotransposition and local gene duplication

The top BLAST hits of predicted KLF18 proteins include several mouse and rat proteins named Zfp352, Zfp353, and Zfp352-like in addition to known KLF proteins. The mRNA of the mouse *Zfp352* gene (NCBI Gene ID: 236537, previously named *2czf48*) was first discovered in a mouse embryonic 2-cell cDNA library [42]. Mouse *Zfp353* (NCBI Gene ID: 234203), with high sequence similarity to *Zfp352*, was later discovered as a gene with expression restricted to lung [15]. The lack of introns inside the coding regions of *Zfp352* and *Zfp353*, coupled with the presence of nearby LINE sequences, raised possibility that these genes are products of two consecutive retrotransposition events [15]. *Zfp352* has an intron in the 5’ untranslated region, while *Zfp353* does not have any introns at all. It was proposed that *Zfp353* is a product of retrotransposition from the mRNA of *Zfp352*, and *Zfp352* is a product of retrotransposition from the mRNA of an unknown gene [15]. Both mouse *Zfp352* and *Zfp353* encode KLF-like proteins with three C-terminal zinc fingers (Figure 2). 

**Figure 5:**
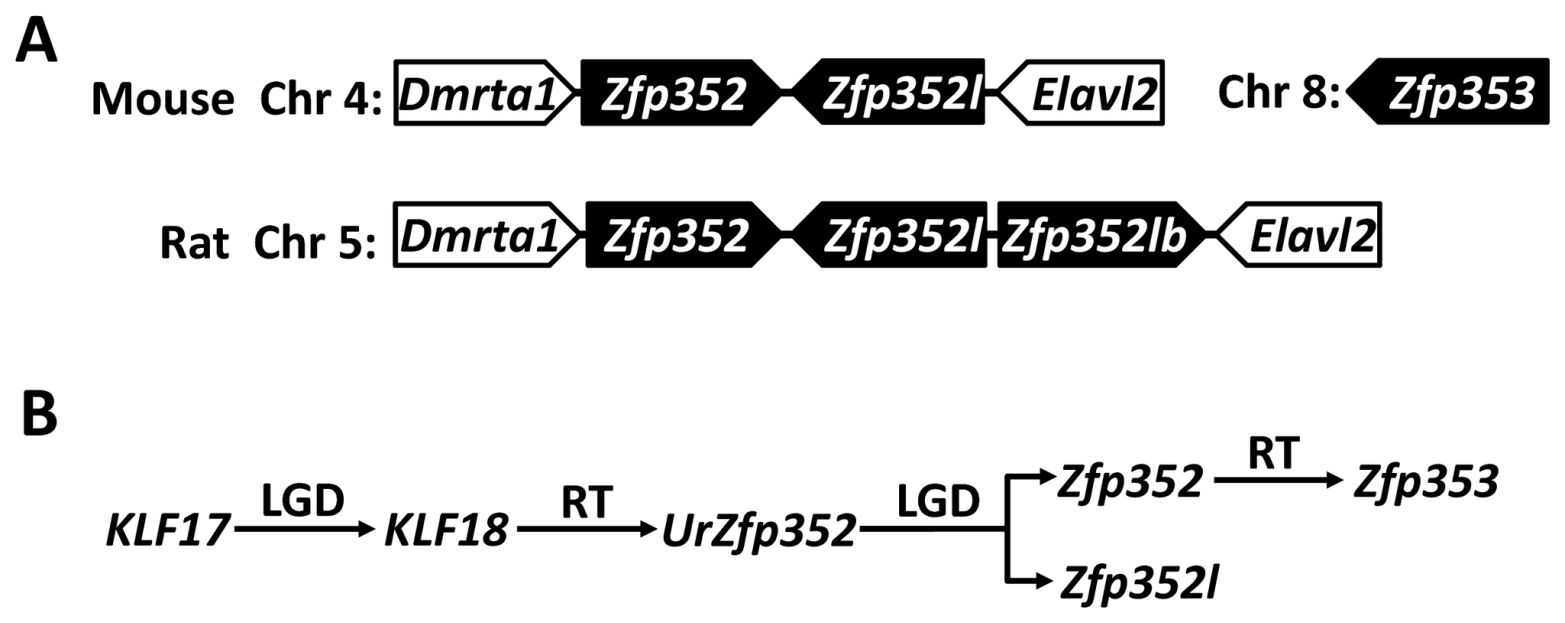
Gene synteny and a model of evolution for *Zfp352*, *Zfp352l*, and *Zfp353*. (A) Gene synteny of *Zfp352*, *Zfp352l*, and *Zfp353* in the mouse and rat genomes. (B) A model of expansion of mouse KLF members. LGD and RT are abbreviations for local gene duplication and retrotransposition, respectively. *UrZfp352* represents the ancestor gene of extant *Zfp352* and *Zfp352l*.

Several close homologs of mouse *Zfp352*, all without introns in coding regions, were also discovered in rat, but not in other mammalian genomes including non-murine rodents. Therefore, it is likely that *Zfp352* originated in the ancestor of the Murinae subfamily. Mouse *Zfp352* and rat *Zfp352* (NCBI Gene ID: 502968) have conserved gene synteny, as both of them are sandwiched by the upstream *Dmrta1* gene and the downstream *Elavl2* gene (Figure 5A). Two predicted genes encoding close homologs of rat *Zfp352* are located near the *Zfp352* gene (Figure 5A). One of them is called *Zfp352l* (NCBI Gene ID: 298232). *Zfp352l* and *Zfp352* are direct neighbors and are oriented in a tail-to-tail fashion (Figure 5A). The other rat predicted gene (named *Zfp352lb* here, NCBI Gene ID: 298233) is a direct neighbor of *Zfp352l* and has the same orientation as *Zfp352* (Figure 5A). Rat *Zfp352l* and *Zfp352lb*, as close homologs of *Zfp352*, are likely generated by local gene duplication events. 

TBLASTN searches using the mouse Zfp352 protein as the query against the mouse genome sequences also revealed a region nearby the mouse *Zfp352* locus that encodes a putative gene. Similar to rat *Zfp352l*, this mouse predicted gene is a direct neighbor of *Zfp352*, and they are arranged in a tail-to-tail fashion (Figure 5A). Therefore, this predicted mouse gene should be an ortholog of the rat gene *Zfp352l* and is thus named mouse *Zfp352l*. Although mouse *Zfp352l* is currently listed as a pseudogene (MGI:3650768, NCBI Gene ID: 619842) in the MGI database and the NCBI gene database, it has evidence of being expressed. Its NCBI UniGene record (Mm.484218) contains one cDNA clone (RIKEN clone 7420403B16, GenBank: AK135677.1) and two ESTs (GenBank: CJ052470.1 and BB706967.1), all of which are from cDNA libraries of fertilized eggs. Interestingly, the mouse *Zfp352* gene was found to be expressed in the two-cell stage of the early embryonic development (cDNA GenBank: AF290196.1; EST GenBank: AA414357.1, AA422810.1, and AI642873.1). These limited expression data suggest that mouse *Zfp352* and *Zfp352l* may encode KLF proteins that function in early embryonic development. We did not find the counterpart of rat *Zfp352lb* in the mouse genome (Figure 5A), suggesting that *Zfp352lb* either has been lost in the mouse genome or is an invention in the rat genome.

Several lines of evidence suggest that *KLF18* is the parent gene that gave rise to *Zfp352*/*Zfp352l* (intronless in coding regions) by retrotransposition of an ancestral spliced *KLF18* mRNA. First, the closest KLF homologs of Zfp352 and Zfp352l are predicted KLF18 proteins. Second, Zfp352 and Zfp352l proteins are grouped with predicted KLF18 proteins in phylogenetic analyses (Figure 4, Figure S3, and Figure S4). Third, predicted KLF18 proteins, Zfp352, and Zfp352l share the repeats containing the common sequence motif [YC]x[sE][QH] that are not found in other KLF proteins (Figure S2). Inference of the ancestral *KLF18* mRNA suggests that *KLF18* was an actively expressed gene (transcribed and spliced to intronless mRNA) in the ancestor of mouse and rat.

Four new KLF gene/pseudogene members were discovered in the mouse genome: *KLF18*, *Zfp352*, *Zfp352l*, and *Zfp353*. Their chromosomal locations (Figure 5A) and gene structures suggest that they originated by local gene duplication (LGD) or retrotransposition (RT) in various stages of evolution since the ancestor of placental mammals. The proposed model of KLF expansion in the mouse genome is illustrated in Figure 5B. In this model, the chromosomally close *Zfp352* and *Zfp352l*, both intronless in their coding regions, are mostly likely the results of a local gene duplication of an ancestral gene, named *UrZfp352*. This ancestral gene *UrZfp352* likely resulted from the restrotransposition of the spliced mRNA of the ancestral *KLF18* gene. *KLF18* itself, being chromosomally close to *KLF17*, is possibly a product of local gene duplication that occurred in the ancestor of placental mammals. *Zfp353*, a mouse-specific gene, is not present in rat. *Zfp353* is not chromosomally close to *Zfp352* (Figure 5A). Its high similarity to *Zfp352* and intronless gene structure suggest that *Zfp353* aroused recently in the ancestor of mouse via retrotransposition of the *Zfp352* mRNA [13] (Figure 5B). 

While the *KLF18*-derived *Zfp352*, *Zfp352l*, and *Zfp353* genes have been found to be expressed in certain tissues such as early embryos and lung [15,43], no expression data have been reported for the predicted *KLF18* genes. The gene or pseudogene status of *KLF18* remains to be experimentally investigated. Our analyses suggest that *KLF18* could still be an active protein-coding gene in some extant mammals, as supported by consistent protein-coding gene predictions by GENSCAN or FGENESH across the majority of available genomes of placental mammals, conservation of zinc finger motifs including zinc-binding and DNA-binding residues, and the favorable score in protein coding potential analysis. Current unavailability of *KLF18* expression data suggests that *KLF18* may perform specialized functions with a tight spatial or temporal expression pattern. In the opposite scenario of *KLF18* being a pseudogene, it represents an interesting case that an ancestrally active parent gene (*KLF18*) gave rise to currently active new genes (*Zfp352*, *Zfp352l*, and *Zfp353*) through retrotransposition, while the parent itself became a pseudogene in extant placental mammals.

## Supporting Information

Table S1 **Predicted *KLF18* genes in mammalian genomes.** Most of the genomes are from UCSC genome browser except a few NCBI genomes not in UCSC or of later version than UCSC genomes. In the "Notes of KLF18 prediction" column, genomes where predicted *KLF18* was not found are marked by red “N/A”, and inferred *KLF18* pseudogenes with stop codon inside zinc finger regions are also marked in red.
(XLSX)

Figure S1 **Alignment of zinc fingers of 36 predicted KLF18 proteins.** Conserved cysteines and histidines involved in metal binding of zinc fingers are on black background. Three conserved DNA base-interacting arginines are shaded in magenta. Three negatively-charged residues interacting with the three arginines are shaded in dark grey. Substitutions in these conserved residues are colored red. Insertions and deletions are highlighted in cyan. Color coding of species names is as follows - black: Euarchontoglires; red: Laurasiatheria; green: Afrotheria; and magenta: Xenarthra.
(PDF)

Figure S2 **Sequences of predicted KLF18 proteins (section 1) and Zfp352/Zfp352l/Zfp353 proteins (section 2).** Repeats matching the regular expression of “[YC]x[GASTDNPE][QH]” (x: a single letter) are highlighted in cyan. Zinc finger regions are highlighted in magenta.
(PDF)

Figure S3 **A phylogenetic tree of representative SP/KLF proteins with the human Wilms’ tumor protein (human_WT1) as an out-group.** This tree was generated by MrBayes. Each protein node is denoted by its species name followed by the protein name.
(PDF)

Figure S4 **A phylogenetic tree of SP/KLF proteins generated by MOLPHY.** Each protein is denoted by its species name abbreviation followed by the protein name. Species name abbreviations are: bt, *Bos taurus* (cow); cf, *Canis familaiaris* (domestic dog); ch, *Choloepus hoffmanni* (two-toed sloth); cj, *Callithrix jacchus* (common marmoset); cp, *Cavia porcellus* (guinea pig); dn, *Dasypus novemicinctus* (nine-banded armadillo); do, *Dipodomys ordii* (kangaroo rat); dr, *Danio rerio* (zebrafish); ec, *Equus caballus* (horse); hs, *Homo sapiens* (human); la, *Loxodonta Africana* (African Savannah elephant); mm, *Mus musculus* (mouse); oc, *Oryctolagus cuniculus* (rabbit); rn, *Rattus norvegicus* (rat); sa, *Sorex araneus* (common shrew); tb, *Tupaia belangeri* (tree shrew); xt, *Xenopus tropicalis* (Western clawed frog).
(PDF)
