# Supplementary material for: A New Family of Predicted Krüppel-Like Factor Genes and Pseudogenes in Placental Mammals
Source: PLoS One. 2013 Nov 7;8(11):e81109. doi: 10.1371/journal.pone.0081109 (PMC3820594; doi:10.1371/journal.pone.0081109)
Supplement: Figure S1 — Alignment of zinc fingers of 36 predicted KLF18 proteins. Conserved cysteines and histidines involved in metal binding of zinc fingers are on black background. Three conserved DNA base-interacting arginines are shaded in magenta. Three negatively-charged residues interacting with the three arginines are shaded in dark grey. Substitutions in these conserved residues are colored red. Insertions and deletions are highlighted in cyan. Color coding of species names is as follows - black: Euarchontoglires; red: Laurasiatheria; green: Afrotheria; and magenta: Xenarthra. (PDF) [file pone.0081109.s002.pdf]

Figure S1. Alignment of zinc fingers of 36 predicted KLF18 proteins. Conserved cysteines and histidines involved in metal binding of zinc fingers are on black background. Three conserved DNA base-interacting arginines are shaded in magenta. Three negatively-charged residues interacting with the three arginines are shaded in dark grey. Substitutions in these conserved residues are colored red. Insertions and deletions are highlighted in cyan. Color coding of species names is as follows: black: Euarchontoglires; red: Laurasiatheria; green: Afrotheria; and magenta: Xenarthra.

```
human              CTYEDCKMSYSKACHLRTHMRKHTGEKPYVCDVEGCTWKFARSDELNRHKKRHTGERPYLCSICSKNFARSDHLKQHA-KVH
chimp              CAYQDCGKSYTKSHHLKDHMRKHTGEKPFVCNAPECEWKFTRLVDLLRHKNKHNRKRSYPCSMCNKSFSRLCYLRQHEKKKH
gorilla            CTYEDCKMSYGKACHLRTHMRKHTGEKPYVCDVEGCTWKFARSDELNRHKKRHTGERPYLCSICSKNFARSDHLKQHA-KVH
orangutan          CTYEDCKMSYSKACHLRIHMRKHTGEKPYVCDVEGCTWKFARSDELNRHKKRHTGERPYLCSICSKNFARSDHLKQHA-KVH
gibbon             CTYEDCKMSYSKACHLRTHMRKHTGEKPYVCDVEGCTWKFARSDELNRHKKRHTGERPYLCSICSKNFARSDHLKQHA-KIH
rhesus             CTYENCKKSYKKACHLRTHMRKHTGEKPYICDVEGCMWKFTRSDELNRHKKRHTGERPYLCSICNKNFARSDHLKQHA-KVH
baboon             CTYENCKKSYKKACHLRTHMRKHTGEKPYICDVEGCTWKFTRSDELNRHKKRHTGERPYLCSICNKNFARSDHLKQHA-KVH
squirrel_monkey    CPHEDCKKSYSKACHLRTHMRKHTGEKPFVCDVEGCTWKFIRSDELSRHKKRHTGERPYLCPVCNKNFARSDHLKQHA-KVH
marmoset           CPHKDCKNSYSKACHLRTHMRKHTGEKPFVCDVEGCTWKFGRSDELNRHKKRHTGERPYLCPVCSKNFARSDHLKQHG-KVH
bushbaby           CAYEDCGKSYAKSYHLRIHQRKHTGEKPYACDVTGCTWRFPRSDELKRHKRKHSGERPYRCMKCRKNFARSDHLRQHE-KMH
tree_shrew         CTYQDCGKSYAKSSHLRIHERKHTGEKPYVCNVMGCTWKFPRSDELSRHKRRHSGERPYLCTDCNRNFARSDHLKQHQ-RVH
mouse              CSKENCGKAFVKSSQLREHERIHTGEKPYICTYYPCTWKFARQDVLARHKRKHTGYRPFKCENCDMTYSRSDHLKAHI-KRC
rat                CHY--CGHSFSKSSHLTGHIRKHTGEKPYKCSR--CTWTFSRSDELTRHMRKHTGDRPHQCQICYTTYPRSDNLNEHV-KKH
kangaroo_rat       CTYPGCGKSYLKPSHLQIHNRKHTGEKPYVCDVTGCTWKFSRSDELKRHKRTHSQDRPYLCTKCNKNFARADHVKQHE-RVH
naked_mole-rat     CPHQDCGKSYARSSCLRVHQRTHSGEKPYACNVQGCEWRFSRSDELKRHMRRHTGERPYKCEACSKSFARSDHLTQHQ-RVH
guinea_pig         CPHQGCGKSYMRPSHLRVHQRTHSGQKTYACNVQGCEWRFNRSDELKRHMRRHSGERPYTCPICQKKFPRSDHVIQHQ-RVH
squirrel           CTYQDCGKCYTKYSHLQIHERKHTGDKPYVCNEIGCTWKFTRSDELRRHKRKHSGERPYLCTRCCKTFARSDHLKQHE-RVH
rabbit             CTYPDCGKSYSKSSYLQIHKRKHTGEKPYECSEEGCPWRFSRVDELSRHKRKHSGERPYTCTKCDKSFARSDHLRQHQ-KVH
pika               CTYPDCGKSYSKPSHLRIHERRHKGERPYACDITGCTWTFSRKDELTRHKRKHSGERPYMCPKCNKTFARSDHVQQHQ-KVH
alpaca             CTYQDCGKSYSKSSHLRIHERLHT----------------------------------------------------------
sheep              CTY-NCGKAYAKSSHLRIHERVHTGEKPYKCNANGCTWAFSRSDELLRHKKRHTRERPYLCTICNKDFARSDHLKQHQ-RVH
cow                CTYKNCGKAYAKSSHLRIHERVHTGEKPYKCNVNGCTWAFSRSSELNRHNKRHTRERPYLCAICDKDFARPDHLTQHQ-RVH
dolphin            CTYQGCGKSYSKSSHLRIHERLHTGEKPYKCNAKGCTWAFSRSDELNRHMRKHTRERPYQCTKCDRNFSRSDHLKQHQ-RVH
horse              CTYQDCGKSYTKPSHLRIHERTHTGEKPYECNVKGCTWKFSRSDELNRHKRKHSGERPYWCTKCHRTFARSDHLKQHE-RVH
cat                CTYQDCGKSYTKPSHLRIHERKHTGEKPYECNVKGCTWKFPRSDELSRHKRKHSGERPYLCTKCNRNFARSDHLKQHQ-RIH
dog                CTYQDCGKSYTKPSHLRIHERKHTGEKPYKCNVKGCTWRFPRSDELNRHKRKHSGERPYLCTKCNRNFARSDHLKQHQ-RIH
panda              CTYQDCGKSYTKPSHLRIHERKHTGEKPYKCNVKGCPWKFPRSDELNRHKRKHSGERPYLCTKCNRNFARSDHLKQHQ-RIH
microbat           CTYQDCQKSYSKKSCLQIHDRKHTGERPYKCNVKGCTWEFARSDELKRHNKKHSGERPYLCTLCDRRFARSDHLKQHQ-KVH
megabat            CTYQDCGKSYSKRSHLQIHERKHTGEKPYKCNWKGCTWEFARSDELNRHKRKHSGERPYLCTLCNKNFARSDHLKQHQ-RVH
shrew              CTFQGCGKQYAKPYQLRIHERVHTGDKPYICDVKGCPWKFARSDELSRHKKKHTGERPYRCPQCPMDFARADHLKQHA-RVH
elephant           CTYQDCGKSYLRRARLRIHECIHTGEKPYICNVKGCARKFSRSDGLYRHKKKHNGERPYVCTRCNKNFARSDHLKLHQ-KSH
manatee            CTYQGCGKSYSRHSRLRIHECIHTGEKPYICNVKGCTWKFSRLDGLNRHKRKHSGERPYLCTTCNKNFARSDHLKLHQ-KSH
elephant_shrew     CPYQGCGKFFSKLSYFQKHQRIHTGMKVFVCNVKGCMQKFCHSDGLGRYKKIHSNERPYLCTVCNKGFSRSAHLKQHF-KTH
cape_golden_mole   CTYQDCEKSYSTHANLRKHEFIHTGKKPYICNVKGCTWKFSRSDGLKRHTKKHSGERPYLCTTCNRNFARSDHLKQHQ-KQH
armadillo          CKHQDCGKSFSKASYIQIHERVHSGEKPYSCDVEGCTWKFTRSDELSRHKRKHSGERPYPCTKCNRSFARSDHLKQHQ-KIH
sloth              CTFQGCGKSFSKPSYLQIHKRVHTGEKPYSCNVEGCTWKFSRTDELSRHKRRHSGERPYPCTKCNRSFARSDHLKQHE-RVH
```
