# Supplementary material for: A New Family of Predicted Krüppel-Like Factor Genes and Pseudogenes in Placental Mammals
Source: PLoS One. 2013 Nov 7;8(11):e81109. doi: 10.1371/journal.pone.0081109 (PMC3820594; doi:10.1371/journal.pone.0081109)
Supplement: Figure S2 — Sequences of predicted KLF18 proteins (section 1) and Zfp352/Zfp352l/Zfp353 proteins (section 2). Repeats matching the regular expression of “[YC]x[GASTDNPE][QH]” (x: a single letter) are highlighted in cyan. Zinc finger regions are highlighted in magenta. (PDF) [file pone.0081109.s003.pdf]

Figure S2. Predicted KLF18 proteins (section 1) and Zfp352/Zfp352l/Zfp353 proteins (section 2). Repeats matching the regular expression of “[YC]x[GASTDNPE][QH]” (x: a single letter) are highlighted in cyan. Zinc finger regions are highlighted in magenta.

Section 1: predicted protein products of KLF18 genes

```
>human chr1_24.16 length=1254
MQPGAGILVLGLLLLPQDAPGLSSVASLNCLSHPRHYDWKHVFWKLSSCWDSGTEKLCEYNKCGKSFCQKESLPTSQKIRTEERPFESKDCGKVLIQKSNFIQYQRMHTEEKPFVYKE
CGKTFSGKSNLTEQEKFHIGEKPFKCSECGTAIGQKKKYLIRNHTGEKSYECKECGNIFCQQIPLIVDDPITKDLVQSSGNIKEMDSSLLQAIEEIEKFFQHLSERHTEQAETPDAPE
PQNCMPLTAHAEESQHESTQSKTMPPLGSTMMTSACTNIPGTVLTQDLTMHPLKALEDLSETYSMGQKVTSFDQVKHTAGSQMTDVTVTPKSSPTDCQKTTITASNMTISNESSQLNT
PSSDQTLNESQIPALLGDQMKTLSDNQTLCGDQVTFSSDQTLTDGHTVTSGSDETLSGGQMTTSLDLYGGQMMTSIDNQTLCGEQMTTSSGNQAFYGRQMTTSTGNQTLCGEQMTTST
GNQALYGGQMTTSASNQTLCGEQMTTSTSNQTLCGEQVMTSTGNQALCGGQMTTSTGNQNLYGGQMMTSTGNQTLYWGQMMTSTGNQNLCGEQVMTSTGNQALCGGQMTTSTGNQNLY
GGQMMTSTGNQTLYWGQMMTSTGNQNLCGEQVMTSTGNQALCGGQMTTSTGNQNLCGEQVMTSTSNQTLCGEQTTTSTSNQTLCGEQVTTSTGNQALYGGQMMTSTGNQTLYWGQMMT
STGNQNLCGEQMTTSTGNQALYGGQMTTSTSNQTLCGEQMTTPTSNQTLCGEQVTTSTGNQALYGGQITTSTSNQTLCGEQMTTSTSNQTLCGEQVTTSTGNQALYGGQMMTSTGNQA
LYGGQMTTSASNQTLCGEQMTTSTSNQTLCEEQVMTSTGNQALCGEQMTTSTGNQALYGGQMTTSTSNQTLCGEQTTTSTSNQTLCGEQVTTSTGNQALYGGQMMTSTGNQTLYWGQM
MTSTGNQNLCGEQMTTSTGNQALYGGQMTTSTSNQTLCGEQMTTPTSNQTLCGEQVTTSTGNQALYRGQITTSTSNQTLCGEQMTTSTSNQTLCGEQVTTSTGNQALYGGQMMTSTGN
QNLYGGQNMTSTDNQALYGGQMATYSGNQTLYGDQMLTLQVGNMTTLTDDHSLYGGYMMSHQFSSLPYPGFLCFSSSHLIQGQLPKQKTQSCQFWKNPEVSRPYVCTYEDCKMSYSKA
CHLRTHMRKHTGEKPYVCDVEGCTWKFARSDELNRHKKRHTGERPYLCSICSKNFARSDHLKQHAKVHNIRPGL
>chimp chr5_22.116 length=529
MESSQLISAQSLEMPTWNQTTMTSVYSSTPKTTSTPNSTALPEHSDFHFGEPSEAASLHQHVVSTDQTQYPTFSHEMELNSASNSRLTHPQMIAVSDIRETAYNEDYWMKTLRSNWCE
SQKVVLGDNLVALCGSQADQDPNEDHLIASACDHYFRGNLEMPSSREQAYYTQMKSRNGGHLTLYETQMITANCSQNFLPNQIINSFSEQTPFGNQHGKAVADEMFSGDQMIASHGYQ
AFYEHQMTDSGDYINCDGEMTSWRNQNVNRGQVKLLGGDNTLYEHQASVYAADQDLCVDHQRSSPIGDQPLYDSQMSTLGFDTTLHVTNREKSNKENLSFHPRTISSSEEAFNWDQMK
TPFVQNVYPDQLGPLNVEESLDPQVTSLSNQTPYVVVSPCPSSSFLAQRQPLESSAASSLIQGQHPKMTSSLKTQNTHHLRPYLCAYQDCGKSYTKSHHLKDHMRKHTGEKPFVCNAP
ECEWKFTRLVDLLRHKNKHNRKRSYPCSMCNKSFSRLCYLRQHEKKKHSQSSPTNGT
>gorilla chr1:45643325-45650824/tmp/12_17_12-00:43:10.fasta|GENSCAN_predicted_peptide_1|1180_aa
DPITKDLVQSSGNIKEMDSSLLQAIEEIEKFFQHLSERHTEQAETPDAPEPENCMPLTARAEESQHESTQSKTMPPLGSTMMTSVCTNIPGTVLTQDLTMHPLKALEDLSDTYSMGQK
VTSFDQIKHTAGSQMTDVTDTPKSSPTDCQKTTITASNMTISSESSQLNTPSSDQTLNESQIPALHGDQMKTLSDNQTLCGDQVTFSSDQTLTDGHTVTSSSDQTLSGGQMTTSLDLY
GGQMMTSIDNQTLCGEQMTTSSGNQALYGRQMTTSTGNQTLCGEQMTTSTGNQALYGGQMTTSTGNQALYGGQMTTSTGNQALYGGQMTTSTGNQTLYGGQMTTSTSNQTLYGGQMTT
STSNQTLCGEQMTTSTSNQTLCGEQLMTSSGNQALYREQMMTSISNQTLCGRQMTTSTGNQTLYGRQMTTSTGNQTLYGRQMTTSTGNQTLCGEQMTTSTGNQALYGGQMTTSASNQT
LCGRQMTTSTGNQTLYERQMTTSTGNQTLCEEQVTTSTGNQALYREQMTTSTSNQTLCGRQMTTSTGNQTLYGRQMTTSTANQTLWGEQMTTSTGNQALYGGQMTTSTSNQTLCGEQT
TTSTSNQTLCGEQVMTSTGNQALCGGQMMTSTGNQNLYGGQMMTSTGNQNLCGEQVTTSTGNQALYGGQMITSTGNQALYGGQMTTSTSNQTLCGEQVTTSTGNQALYGGQMTTSTSN
QTLCGEQVTTSTGNQALYGGQMMTSTGNQTLYWGQMMTSTGNQNLCGEQMTTSTGNQDLYGGQMTTSTSNQTLCGEQMTTSTSNQTLCGEQVTTSTGNQALYGGQMMTSTGNQTLYGG
QMTTSTSNQTFCGEQVTTSTGNQALYGGQMTTSTSNQTLCGEQMTTSTSNQTLCGEQVTTSTGNQALYGGQMTTSTGNQALYGGQMMTSTGNQALYGRQMTTSTSNQALRGEQVMTST
GNQALYGGQMTTSASNQTLCGEQMTTSTSNQTLCEEQVMTSTGNQNLYGGQNMTSTDNQALYGGQMATYSGNWTLYGDQMLTLQVGNMTTLTDDHSLYGGYMMSHQFSSLPYPGFLCF
SSSHLIEGQLPKQKTQSCQFWKNPEVSRPYVCTYEDCKMSYGKACHLRTHMRKHTGEKPYVCDVEGCTWKFARSDELNRHKKRHTGERPYLCSICSKNFARSDHLKQHAKVHNIRPGL
>orangutan chr1.3726 length=655
MDSSLLQAIEEIENFFQHLSEQHAEQAETPDAPEPQNCMPLTAHAEESQHESTQSKMMPPLGSTMMTSACTNTPGTVLTQDLTMCALKALEDLSETYSMGQKVTSFDDQIKHTAGSQM
TDVTDTPKSSPTDCQKTTITASNITVSSESSQLNTPSSDQTLNENQTPALHGDQMKTLSDNQTLCGDQVTFSSDQTLTDGHTVTSSSDETLSGGQMTTSLDLYGGQKMTSIDNQTLCG
EQMTTSSGHQALCGEQVTTSTGNQTLYGGQMTTSTGNQALYGEQVTTSTSNQTICGEQVTTSISNQTFCGEQTLHGEQMTTSTGNQTLYREQMMTSTSNQTHCGEQMTTSTGNQNLYR
EQMMTSTSNQTLCGEQMTTSPGNQALYGGQMTTSTSNQALYGGQMTTSTSNQTLYGGQMTTSTSNQTLCGEQMMTSTSNQTLYGGQMMTSTGNQTLYGGQNMMTSTFNQNLYGGQNMT
PIDNQAVYGGQMATYSGNPTLYGDQMLTLQVGNMTTLTDDHSLYGGYMMPHQFLSLPYPGFLCFSSSHLIQGQLPEKQKTQSCQFWKNPEVSRPYVCTYEDCKMSYSKACHLRIHMRK
HTGEKPYVCDVEGCTWKFARSDELNRHKKRHTGERPYLCSICSKNFARSDHLKQHAKVHNIRPGL
>gibbon GL397442.25_prot length=810
MDQFLDPITKDLVQSSGNSKEMDSSLLQAIEEIENFFQRLSERHTEQAKTPDAPEPKNCMPLTAHAEESQHESIQSKTMPPLGPTMMTSACTNIPGTVLAQDLTMHPLKALEDLSETY
SMGQKVTSFDQIKHTAGSQMTDVTDIPKSSPTDCQKTAITASNTTISNESSQLNTPSSDQTLNESQIPALHGDQMKTLSDNQTLCGDQVPFSSDQTLTDGHTVISSSDEILSGGQMTS
LNLYGGQMMTSIDNQTLCGEQMTTSSGNQALYREQMTTSTGNQTLCGEQVMTSTGNHMLYREQVTTSTGNQTLYGGQMTTSTSNQTLYGEQMTTSTGNQTLYGGQMTTSSDNQTLYGG
QMTTSTGNQTLYGEQMTTSTSNQTLYGGQMTTSSDNQTLYGGQMTTSGDNQTLYGGQMTTSTSNQTLYGEQMMTSGDNQTLYGGQMTTSTSNQTLYGEQMMTSSDNQTLYGGQMTTST
SNQTLYGEQMMTSSDNQTLYGGQMTTSSDNQTLYGEQMMTSTSNQTLYGGQNMMTSTSNQTLYGGQMTTSSDNQTLYGGQMMTSTSNQTLYGGQNMMTSTSNQTLYGGQMMTSTSNQT
LYRGQMMTSTGNQTLYGGQNMMTSTSNQNLYGGQNMNSIDNQALYGGQMATYSGNSTLSGDQMLTLQVGNMTTLTDDHSLYGGYMMSHPFSSLPYPGFLCSSSSHLIQGQLPEKQKTQ
SCQFWKNPEVSRPYVCTYEDCKMSYSKACHLRTHMRKHTGEKPYVCDVEGCTWKFARSDELNRHKKRHTGERPYLCSICSKNFARSDHLKQHAKIHNIRPGL
>rhesus chr1.1186_prot length=671
MMTSAYTNIPGTVLTQDLTMHPLKALDMALEDLNETYSMGQKVTSFDQIKHTAGSQMTDVTDTPKSSPTDCQKTAITASNMTISSESNQLNTPSSDQTLNESQIPALHGDQMKTLSDN
QTLCGDQVTFSSDQTLTDGHTVTSSGDEALSGGQMTTSLDLYGGKMMTSTDNQTLYGEQATTSTGNQTLYGEQMMPSTSNQTLYGEQMMPSTSNQTLYGEQMMPSTSNQTLYGEQMMP
STSNQTLYAEQMTPSTSNQTLYGEQMMPSTNPLRGAGDEQMTTSTGNQTLYGEQATTSTSNQTFYGEQMTTSTGNQTLYGEQMMTSTSNQTLYGEQATTSTSNQTFYGEQMTTSTSNQ
TLYGEQMTTSTGNQTLCGEQMTTSTGNQTLCGEQMTTSTGNQALYGGQMMTSTSNQTLCGEQMTTSTSNQTLCGEQMTTSTSNQTLCGEQMTTSTSNQTLCGEQMMTSTSNQTLCGEQ
VTTSTGNQTLYGGQNMTSIDNQALCGGQMATYSGNPTLYGDQMLTLQVGNMTMLTNDHSLYGGYISHQFSSLPHPGFPCFSSSHLIQGQVPEKQKTQSCQFWKNPEVSRPYICTYENC
KKSYKKACHLRTHMRKHTGEKPYICDVEGCMWKFTRSDELNRHKKRHTGERPYLCSICNKNFARSDHLKQHAKVHNVHPGL
>baboon scaffold14273.2_prot length=1390
MDSSLLQAIEEIENFFQHLSERHTKQAETPDAPEPQNCMPLTAHAEESQHESTRSKTMPPLGPTMMTSACTNIPGTVLTQDLTMHPLKALDMALEDLNETYSMGQKVTSFDQIKHTAG
SQMTDVTDTPKASPTDCQKTAITASNMTISSESNQLNTPSSDQTLNESQIPALHGDQMKTLSDNQTLCGDQVTFSSDQTLTDGHTVTSSGDEALSGGQMTTSLDLYGGQMMTSTDNQT
LYGEQATTSTGNQTLYVEQMTTSTGNQTLCGEQMMTSTGNQTLYVEQMTTSTGNQTLCGEQMMTSTGNQTLYAEQMTTSTGNQTLYGEQMTTSTGNQTLYGEQMTTSTGNQTLYGEQM
MTSTSNQTLYGEQMTTSTSNQTLYREQMTTSTSNQTLYGEQVTTSTGNQTLYGEQMTTSTGNQTLYGEQMMTSTSNQTLYGEQMTTSTSNQTLYREQMTTSTSNQTLYGEQVTTSTGN
QTLYGEQVTTSTGNQTLCGEQMTTSPGNQTLCGEQMTTSTGNQTLCGEQMTTSTGNQALYGGQMMTSTSNQTLYGEQVTTSTSNQTLYGEQMTTSTGNQTLYGEQMMTSTSNQTLYGE
QMTTSTSNQTLCGEQMMTSTSNQTLYGEQMTTSTGNQTLYGEQMMTSTSNQTLYGEQMTTSTGNQTLYGEQMTTSTSNQTLCGEQMMTSTGKMTSTGNQTLYREQMTTSTSNQTLYRE
QMTTSTSNQTLYGEQMTTSTGNQTLCGEQMTTSTGNQTLCGEQMTTSTGNQALYGGQMMTSTSNQTLYGEQVTTSTSNQTLYGEQVTTSTGDQTLCGEQMTTSTGNQTLCGEQMTTST
GNQALYGGQMMTSTSNQTLYGEQAMTSTGNQTLYGEQTLYREQMTTSTSNQTLYGEQVTTSTGNQTLYGEQVTTSTGNQTLCGEQMTTSPGNQTLCGEQMTTSTGNQTLCGEQMTTST
GNQALYGGQMMTSTSNQTLYGEQAMTSTGNQTLYGEQVTTSTSNQTLCGEQMMTSTSNQTLCGEQMTTSTSNQTLYGEQVTTSTSNQTLYGEQATTSTSNQTLYGEQATTSTGNQTLY
GEQATTSTGNQTLYGEQVTTSTGNQTLCGEQMMTSTSNQTLYGEQVTTSTGNQTLYGEQVTTSTGNQTLYGEQVTTSTSNQTLCGEQMMTSTGNQTLSGEQVTTSTSNQTLCGEQMTT
STSNQTLYGEQVTTSTGNQTLYGGQNMTSIDNQALCGGQMATYSGNPTLYGDQMLTLQVGNMTMLTNDHSLYGGYISHQFSSLPHPGFPCFSSSHLIQGQVPEKQKTQSCQFWKNPEV
SRPYICTYENCKKSYKKACHLRTHMRKHTGEKPYICDVEGCTWKFTRSDELNRHKKRHTGERPYLCSICNKNFARSDHLKQHAKVHNVHPGL
>squirrel_monkey JH378193.76_prot length=378
MEVYDATPMSQRALCGEQVTTSTGNQALCGGQVTTSTGNQALCGGQVTTSTGNQALYGGQVLIYTGNQALCGSNMAIYSDNSTLSGGHMLTLQAGNMTTLTGDHSLYGGYMTSCQPSL
SYPGFLCFSSSHLIQGQVSEKQKTQSSQFWKNPKISKPFICPHEDCKKSYSKACHLRTHMRKHTGEKPFVCDVEGCTWKFIRSDELSRHKKRHTGERPYLCPVC
NKNFARSDHLKQHAKVHNICPEL
>marmoset chr7.8.132.a length=866
MPVARSWVCRKTYVTPRRPFEKPRLDQELKLISEYGLRNKREVWRVKFTLAKIRKAAGELLTLDEKDPRRLFEGNTLLRWLVRIGVLDEGKMKLDYILGLKIEDFLERRLQTQVFKLG
LAKSIHHARVLIRQRHIRVRKQDPITKDIVQSSANSKEMDSSLLQAIEEIKNFFQHLSEQHTKQAETPDAPEPQCCMPLPAHAEESRHESTQSKTMPPLELTKMTSACTNIPGTVPTQ
DLTVHFLKALDTALEGLSETSSMGQKVTSFDQIKHTAGSQMTDVTDTPKTSLTDCQKTTVTASNMAISSEGSQLKILSSDQTLSESQTQALRGDQMKTLSDNQTLYRDQMTFSSDQTL
```

```
TDGHTVTSSGDRALSEGQMITSLDLYGGQMMTSIDNQTLCGEQTTTSTPRQALYQGQVTTSTGNQALCGEQTMTSTDNQALFGEPTTTSTDNQTLCGEQTMTSTDNQILYGGQMTTCT
GNQALFGEQMTTSTGNQALYGGQMTTSTGNQALYGEQMTTPTGNQALYGEQMTTSTGNQALCGGQMTTSTGNQALCGGQMTTSTGNQALYGGQMTTSTGNQALYGGQMTTSTGNQALY
GGQMTTSTGNQALYGGQMTTSTGNQALYGGQMTTSTGNQALYGGQMTTSTGNQALYGGQMTTSTGNQALYGGQMTTSTGNQALYGGQMTTSTGNQALYGGQNMTSIDNQALHGGHMAI
YSDNLTLYGGHMLTLQAGNMTTLTGDHSFYGSSLSYPGFLCFSSSHLIQGQLSEKQKTQSSPFWKNPKPFICPHKDCKNSYSKACHLRTHMRKHTGEKPFVCDVEGCTWKFGRSDELN
RHKKRHTGERPYLCPVCSKNFARSDHLKQHGKVHNICPEL
>mouse_lemur scaffold_116910.1_prot length=345 (zinc finger region not detected in current genome with low coverage)
DPTLEEIIQFLYNSKEMDFNCFQSLDDIDALLQDLSEPQKEQAKTPGAPEPQPNVPLNAHGEHNPHESTQSQEMPPVRLTMTSVCSDIPGRVLTQNSTPPPVKALDIPIGDLHKTFLM
GQKVTSCDQIKHIAGSQMTDVTDTPKIPVADCQKTTITANKIAISNEGNQKTLSSDQTLSGSQMPTQMKTLSNNHTLSTGQMIFNGDQTFYGGQMTPSGDQTIYGDQMTTLSVNQTRR
YTMTSSGDQTLSGSQMVTFNANQTLTGKYMTSNGDQTLYGDQMVTYSGDQTLYGGQMVTFNANHILTGRYTMTSSGDQPLYGDQMVTYSGDQTLYGGQMMTVSGDQTLK
>bushbaby GL873721.26_prot length=419
MDPTNEDLIQSLGNSKEMDSSVLQMFEEIDKFFQGFSEPHEKQTNPLGAPELTAQGEYSQHESTQSVVVPPWRPTVMTSVSSDMPGTVLTQNSTTLPVKGTDTPFGDLSKTFSIGQKM
TSGEQIEYTASSQMTDVTDTPKTTVTNYQITTMAANKIAISSECDQTVRGSQVTSLYEDWMKALGDGQTCFGGQMTSSGDQTYYEDQKTPFIGHQSHYWDKMTTSFADQTSDGDQMAT
FSGDQIFAEYHAVPSSSGQTLYGDQMMAFSGDQAIRGGHTRNYSTDQTLYGSYSQFHQSSLPYSRSLDFSSSHLMQEQLPEKQKPYICAYEDCGKSYAKSYHLRIHQRKHTGEKPYAC
DVTGCTWRFPRSDELKRHKRKHSGERPYRCMKCRKNFARSDHLRQHEKMHS
>tree_shrew scaffold_121725.1-22529.2_prot length=454
MSLGNSKETYSYVQTIEEIKEFLQDLTEPYKEQAKTPAVLEPQPYMPPTAPGDDSQHESNHNQVMPPLRPSVVTSEGSKIPGPVLIQNATTPPVKTLDVPLGDLSETFPIGPKETFVD
QIKPTGSQMIDVTDASRMVFTDCQKTAVLANARAISNEGGQMETLNYDDLSRSQVAVLYGNQVKTSGDNQAFHQSQITTLERGHVMTFIGDQTLTEDHMTFSGDWMTTLKEGQMTLSS
GNQTLYRSHMTITGGNQILRGGQMMTFKGDHMTNFADDHTPYGDHMVSYPPLSLPYPGFLYFPTSHVIEERLAEKQKQNLKTQSCQFQKNSDILRPYICTYQDCGKSYAKSSHLRIHE
RKHTGEKPYVCNVMGCTWKFPRSDELSRHKRRHSGERPYLCTDCNRNFARSDHLKQHQRVHR
>mouse gi|148698598|gb|EDL30545.1| mCG120027 [Mus musculus]
MTSNSSTVTTSEYSTLPQPVLTTHPAKALNIPLAEPAQAFPVNQETLPIDQVIFTASSQLTNVNDSSRPLLPGYQKTAASRNKVTISNEENQITTLDDNRLATLNQGPVVNFNADQTS
IENCPVISGGDQASSWGQMVTFNADQTHHGHQKMTFSSDQNVNRSQVTTSSSDQTMYRDQRTTYSAQNIYRDSMKILSDCYTYYNDQITVPNRNQTFYRGQTMTHDSNLTIYGGQRMP
HDSNLILYGSQTMPLDTNQIPYGGQMTIQTDDLTYGTPPNSYQTFYGSQMATPSADQTVYGYQKMPLSENQALHRSQKTTPSDEPTFYGNQMTPSDGNQSLYGGQRTTYNRNQTYEGQ
TMIPSSNVSYYRAQGTGVQTLCGSQKTTSNDDQTFYGYHMTSPDGNLTFYGGQTTTPNGDQIFYDMQMTTPSGDQTYNTGWMLPCVSSQMPSDDRFSYFSKSHLAPGKPSASPSSFLL
GPGYSPKRMKDLRTPAPPKRKAYICSKENCGKAFVKSSQLREHERIHTGEKPYICTYYPCTWKFARQDVLARHKRKHTGYRPFKCENCDMTYSRSDHLKAHIKRCPSKFQPAPGSL
>rat FGENESH1:   1   2 exon (s)    899  -   3660   691 aa, chain +
MTSCSSTVMTSESSSVPHTVTTMHPATALGKPPAQPHKAVPMSQEMVPVDIMLSTASTQLTHGQDSSKSLLTAYQKTAATGTKVTSPIEDNQINTIYDNLLATLTEGPVVSFSGYQSS
FEDCSVISEGNQASDWGQVVTSNANQTYHGHQTMTFNSEQKVNRNQVTTAIGDQTVYRNQVKTYNDQNVYRDKVKILSANQAYYNNQMTVPNGNQTLYGGQPLPFDTNQTLYTGQPMP
QDPNHTVSGGQTMPFDNIQSLYRGQTMAHKSKQSHSGYQIMSFDTNQTLCEGQAMAHDYNQIRYGGQTIPHDSNQTLYGGHKMPSDTNQNLYGGQTVPFDTNQTLFGGQPLPFDTNQT
LYRGQPMLRDLNHTVSGVQTMPFDNNQSLYRGQTMAHKSKQTHSGDQTVSFDSNQVLHRNQKNTASDETTFYRKQAVPSDGNPILYGDQTTSHFGDQTLFKGQPMNPSSHYDGAQITG
FQTLSGNQKMTPSDDHQNFYRYYMTATEGNPTFDGDQTTTTNGDQTCNTGWMMSLVSSQMPHGDTVLDFSISHMVQGQTSACSSDFPVVPGNLPHRKNGLRTPTQNTGKAYICHYCGH
SFSKSSHLTGHIRKHTGEKPYKCSRCTWTFSRSDELTRHMRKHTGDRPHQCQICYTTYPRSDNLNEHVKKHHPWVLEMAQWLVEQSSRPDFLSSISQNLFI
>kangaroo_rat scaffold_5409.4_prot length=650
MPDPGGVNSKEMSTFLETLEDMEESLESFSNPYRDYANISTVLESHPSIPLTDFGESNWHSPTHSQVMPSPALGGEQNINGGQTPASTADQTTNGRLMPIPGGDQTINGEQMPLPSGD
QTMYGGQMTVPNRDGTINGGQMPVPGGDQTISEGFMPVPGGDPAIYGGQMPDPSGDHTINGGQIPIPNIEQTINMGQLPAPSGDQIIYGGLMPTPGGEHTMKEEQMPLPSGDQTMYGG
QMSVPNRDGAISGGQMPVPGGDQTISEGLMPVPGGDPTIYGGQMPDPSGDHSTNGGQIPIPSFGQTINMGQLPTPGGEQTINWGQMPIPGGDQTIYGELMPTPVGEQTIKGSQMPVPG
DNQTVNGEQMPVSGVDQTMYGGQMSVPGGDQAVSGGQMPVPGGDQTINVGQMPIPTGVQTIYGRLMPVPGGDQTIYEELMPLPGAEQTINGGQMPIPGSVQTMYGAQMPIPSGVQTLC
GGQMPVPSGVQTSLEEQTMAFLAKHAFGSGHVVTCNSTQNLSWDSVPTQQLLSLTCPQLVHVSRSPSTQGQAPDKWALKTQSFQLPQHPARLKPYVCTYPGCGKSYLKPSHLQIHNRK
HTGEKPYVCDVTGCTWKFSRSDELKRHKRTHSQDRPYLCTKCNKNFARADHVKQHERVHS
>naked_mole-rat JH602205.102_prot length=201
MEDNQDTFTEGQHYKKREYRDPTAKDLLQSCSNSREMNPAFETTEEIDDFLQVFSDPYNEHRQGQPWPEPQLGMAATASAEGSQQGSSQSQAKSSVFLEKPESWKPYMCPHQDCGKSY
ARSSCLRVHQRTHSGEKPYACNVQGCEWRFSRSDELKRHMRRHTGERPYKCEACSKSFARSDHLTQHQRVHRTFPLGLEPNHL
>guinea_pig scaffold_2590.1 length=414
DPRAENTLESSGRSMMNPCFETLEEIDDFLRNFADPYREHEKAQCIPEPHLCMPPTAAGESCQQGSSQSQAMLRCSSAEWMGGGSSGIPGGVHTQNAATAPHESLEGILEELNELFDQ
KAHAMTHEQNWHSAGCQKTPCPGTPQISLPDCRKTHGTASKLTVPEGGRPVQDTRGHQTLNGTQAGALWGGPIISLQDNQTPSGALMTVPLAPQTVPLGATANAICDQSLCGKQWTNT
SSGQRPRGEQPVTLTNGRNPSGGYQLVPAPRLGLVDLPGIPGIQEQAPAQNRSVQMKTYPLWVNSQRPRPYVCPHQGCGKSYMRPSHLRVHQRTHSGQKTYACNVQGCEWRFNRSDEL
KRHMRRHSGERPYTCPICQKKFPRSDHVIQHQRVHK
>squirrel JH393284.830_prot length=614
MGSQERITALSGGASGAALDVRSAALDVHSAALDVHSPRRAQASLGLGLGWNSGSTSENTFHQTTWVCTGQCRKHLKASGGQSRGLDCPQESQTVARCWASANVVTIEEIEELLQVFT
ESYKDMEKMSAVPEPQLYMPLTISGEGRLHEFTQGQSMPSSRPSMETSVCSNIPGMVLTQQTPPSEALDTFLGELSGTLSMNPQMTSFDQIKSSAEFQMMNIADTPETSLIDCQKTTV
TASMMTISSEVSQKKTLIGNQTLSEIQMTLSENQTLYGSQPTTLKKGHVMTFRGFQTLIEDYTMTSSGDQTLCGGQMTMASGNQILYRCQMTTPSGDQTYSGGPMANLNSNQTLYGGQ
MTTPGGDQTFYGSQMATPGGDQTLYRGQMATPGDDQTLYGGQMATPSDDQTLYGGQMATPSDDQTLYGGQIATPGGDQTLYGDQMAISSGDQTLYGDQMAISSGDQTLYGDQMAIPGG
DPTLYRGQMMPHQSSSIPYPGFLNDSSSPLIQGQFPENKWKLKTQRYQFQKNSNILKPYACTYQDCGKCYTKYSHLQIHERKHTGDKPYVCNEIGCTWKFTRSDELRRHKRKHSGERP
YLCTRCCKTFARSDHLKQHERVHR
>rabbit gi|291415738|ref|XP_002724106.1| PREDICTED: mCG120027-like [Oryctolagus cuniculus]
MPTLTPTVPSERPNIPGTVLIQNSTQPSVTDLDRPLEDQSKTLFTDQEVTFDQTKPTADSQMTGVTDIPKMTPTDGQKSAGTAGKLTVSSEDGQLKTLGMIQMTWDDQSNTPSENQTL
HGAQMTTLTERHVRTPGGDQILDGAQKSNTTGGQRFYRGQISNSTDDQTLYGGQMSAPSCDHQTLYGGQMNTPASDQNMYVHQMNSPTGDQTLYGDQVNLPIGDQTLYGGQMNAPSGD
HQTLYGGQMKTPASDQTQYVGQMNSPAGDQTLYGGQVNSPIGDQTLYGGQMNLTTADQTLYGSQMNAPSGDHQTLYGGQMNTPASDQTLYVGQMNSPSGDQNLYQGQLNATTNDQTLF
VGQMHSPTDDQTLYGGQMNVPTANQILYGGQMNTPAIDQTLYVGQMNTPASDQTLYVGQMNSPTGDQTLYQGQLNATTNDQTLFVGQMHSPTDDQTLYEGQMNVPPANQTLYGGQMNT
PASDQTLYVAQMNSPTGDKTLYVGQINLPTGDQTLYGGQMMPFTGNCSLSGGQMMTNSSQVYGTQMMPHQSFSLPHPGFVYASSFLLIQGQPPEYFSSSSLVQGQLLEKQNWNLQTQR
YQPPKNPELLKPYVCTYPDCGKSYSKSSYLQIHKRKHTGEKPYECSEEGCPWRFSRVDELSRHKRKHSGERPYTCTKCDKSFARSDHLRQHQKVHR
>pika scaffold_10881.2_prot length=793
MRAQGPRIWAVLHGFPKPQTGRQTRSGDQNQGPEGILNPTTEDIFLSSGNSEDMSSFPVTAEDSECAQDTSSAQKEQTQMSAVPQPQLHMPPAACDESSWHVSMQSLGIPSLTPTIMT
SAPCDSLLDILLSQDSTALPVAALNTPLGVPKQTVCMDQMLSSWDQTKPSAGSQRMGGMDIAETCPSHCEKTDATAVSTAIPSEGGPLKTLQSRQMTSDGHINGSSDQQSLCEAPMTS
LQERPGLGPGGDQTLSVGQVVTSTSPQSLSLGQMMPSTVDPQLCGMQMNPTTEDIFLSSGNSEDMSSFPVTAEDSECAQDTSSAQKEQTQMSAVPQPQLHMPPAACDESSWHVSMQSL
GIPSLTPTIMTSAPCDSLLDILLSQDSTALPVAALNTPLGVPKQTVCMDQMLSSWDQTKPSAGSQRTGGMDIAETCPSHCEKTDAAAFSTAIPSEGGPLKTLQSRQMTSDGHINGSSD
QQSLCEAPMTSLQERPGLGPGGDQTLSVGQVVTSTSPQSLSLGQMMRSTVDPQLCGMQMVSPMCGQTLYADQAFPPKADQTLCVGQMMMSTPCAQTLYGGQVMTPSIDQIPTGIQMMT
PSIGQTLHGGQMMTTTGYQTFSGGHVVSPTGSQNLYGVQMVMPGGDQTLQGAYLMQPASYSGPHPGCLSVSSYPWIQAQTPQCFSSSYLVQGQLPAQQDCNLPTTQHSQFQKNPERLK
PYICTYPDCGKSYSKPSHLRIHERRHKGERPYACDITGCTWTFSRKDELTRHKRKHSGERPYMCPKCNKTFARSDHVQQHQKVHK
>alpaca scaffold_44452.1_prot length=343
MITHDEDSQKVSLQNQVTASDERKPTATSQMTDVTDTPKTLFTDGQDTMINANKMTIPKEGSQVKTFSEGQTPYGVQMTFSGDQTLYGGQMKTTGEYQTLHGGQMTFSGDQTLYGGQM
KTTGVYQTLHGGQMTFSEDQTLYGGQMNTISECQTLTGGQMIVSGDQTLYGGQMKTTGEYQTLHGGQMTFSGSQTLFGGQMNTVNEYQTLSGGQMTFSGGQTLYMCQIKTTSECQTLS
GGLMPFSGDQTLQGCTMKTLSDDHTLLGSRMMPHSLSSLPYPGSSYISSSHLIQGQSLENQKWNLKTRRCQLQKNSDILKPYTCTYQDCGKSYSKSSHLRIHERLHT
>sheep /tmp/11_28_12-18:22:00.fasta|GENSCAN_predicted_peptide_2|554_aa; predicted from DNA
sequence:gi|406506174|gb|AMGL01001145.1| Ovis aries breed Texel contig_1148, whole genome shotgun sequence; however,
the assembled genome sequence has problems with frameshift in the last exon
MMDTSMDFTTEDQVYWSGNSEESSRDSYLETIEELEEFLQKLDELYGTRSPAAPEPKPCVPPTAHEEDSQKESMQNEVMNLLIHNDDLRMFEHPWDTFSAELTSTHVKALDLPPGNIW
FKHGFCFWGRCVNQTRYCVWTSIDDPTTEDLVQVSGNSKEVSRYSYLQTVEEIKAFLQEANETHKARTPAAPQPLTACEEDDRKESMQRQKPTTEDLVQLSGTSKEASTASFFVTLED
IEEFLHEINEIHEAKTPPAPESELCVPLTAHGDVGQMESVNSQVTSFDERNPTGTSQLMDVTDTPTTFITLCQVTVVNANKRTISNEGSEVGTLSDDQSLCGGQMTISGDQALYGGQM
KSPTVCQNLSRTQMTFAADKTICGNQMETMDEHQTLSVGQMNFSGDQTLYGGQMKTVNDDTLHEAHIMPQSSSSLSYPRTLYVASSHLIQGQPVEMQKWNPKIQRCPLQKRPDILRPY
ICTYNCGKAYAKSSHLRIHERVHTGEKPYKCNANGCTWAFSRSDELLRHKKRHTRERPYLCTICNKDFARSDHLKQHQRVHR
```

>cow chr3.2218_prot length=440
MPSPKSMLKDAEMMAQILKDMEITEYEGVANQRKLVWDCSTSIPGSIKGQVLRPVSQSWKLLAHDAEVPEHMLDAPMDSTTEAQLSGNSQEASRDSYLEAMEELEAFFQELNDTYGTE
TSAVPEAELCVPLTAHEEDNQKEPMQNQKPTTEDLVQLSGNSKEASMTSYFPTLEDIEEFLQEVNETHKTRSPAAPESDVCVPLTAHDDVSQKESLQKQVTSFDERNPAAASRLMDVT
DTLKTFITDCQATAVNANKLTVPSENSQMKTLSDDQSLYGGQMTFSGVQTAYLGEMKKLSDEDTPHRVYMMPLSSASLLYPRILYVASSHLIQGQPVETQKWNLKIQRCPLQKRPDIL
RPYICTYKNCGKAYAKSSHLRIHERVHTGEKPYKCNVNGCTWAFSRSSELNRHNKRHTRERPYLCAICDKDFARPDHLTQHQRVHR
>dolphin FGENESH JH472540 + 85560 88612
MDAAPNQDPTAEELLLLSGNRKKASRDSYLQTIEEIGAFPQDLNENPTTEDLVQSSGNSKEASTNSFFPTLEGIEEFLQDINETYDTRTPAAPEPELYVPLTARDEDSQKESIQNEVT
SSQGRNPTVASQMTDGSDIPKTLITDCQATDANANKMTIPIEGSQMKTLNDNQSRYGGKITFSEDQTLYGGQPLEKQNWNLKIQRCTLQKNSDILKPYICTYQGCGKSYSKSSHLRIH
ERLHTGEKPYKCNAKGCTWAFSRSDELNRHMRKHTRERPYQCTKCDRNFSRSDHLKQHQRVHR
>horse |GENSCAN_predicted_peptide_1|625_aa
MANTVSNVGDTDLISAAGMVHGVEAKEEGSAVGVVGRTSTKGLAGSARVSRERPSKVRSKPSVLFRDSPAGDTGKNNPSAREILIMENGRIRKSHRKKLDPTIEDLLQLSGNSKEIST
DSFLQTLEEIEECLQDFSETHKARTPAVPEPQLYIPPTACGEDSQRESTQSQVMTSLKPTMMISACSSIPGIVLVQYSMPPPVKALDMPLGDLSEIFPMEQVISFDQRKPTANSQMTD
VTDTPKTSFTDCQKITITASKMTIPNECSQMKTLSDDQTLFGGQMTFSGDQILTEHHTVTSGCDQTLNGREMTTLCEEQIHTLSDDQVLYKGQMKTLSGDQTLYGGQMTFSGDQTLYG
GQMTFNGDQTLYGGQMTFSGDQILHGGQMKALNDDQNFYSSQMTFVGDQTLHGGQMKTLKPRWKMTALKGGFTMTFVGNQTFTGSQMTTYSGDQTFCGGQVMTFSCDQTLYGGQMMPL
KRDYMTAFPDDHTLSGGHMMPHQSSSLPYPGFLYFSNSHLIQGQSLEKQKWNLKSRRHQFQKNSDILKPYACTYQDCGKSYTKPSHLRIHERTHTGEKPYECNVKGCTWKFSRSDELN
RHKRKHSGERPYWCTKCHRTFARSDHLKQHERVHR
>cat /tmp/05_29_13-19:21:37.fasta|GENSCAN_predicted_peptide_1|465_aa
MGALGPAPKGVANQRRESAGPRASGLAVYEARRHDSTLLGHALEPLMDPTAEDPVQLLSNSKETSVDSFLQTVEEIEEFLKDVSETYKAKMSAAPEPQLHIPLADRGEDSQHESTQRM
MTPLKSTMVSSTCPNIPGTVIVQNSTIPTVTALDMPPGDLTESFSVIQKVTFFDERRPTTSQMTDVIDNPKTTFTACQKITANKIAIPNEGSQMKTLSDDQILYGGQMTVPKENHMMT
FSAKQTFTEGHTMTSSGNQTLNWNQMTTLCEEQQMKTLSDDQTLYGDHMTFTGGQTLYGGQMETYSGDQMLYGGHMTLSGYQTLYGGQMKSLSDDQTLYGGQMTYSGDQILYDSQVNT
FGDDQTLYGGQMMTLKGHHMTTSTDDHTVYGDHMMPHQSSSLPYPGFLYFSSSHLIYGQTLEKQKCNVKTQRCQVQKNPNVLKTYTCTYQDCGKSYTKPSHLRIHERKHTGEKPYECN
VKGCTWKFPRSDELSRHKRKHSGERPYLCTKCNRNFARSDHLKQHQRIHR
>dog chr15:16115394-16138013FGENESH: 1 3 exon (s) 11061 - 18219 535 aa, chain +
MRGPGRATRRAPGGAAGAASQDPMTEDLVQSSCNSRETSMDSLPQTIEEIEEFLKEVSESYKARTAAAPEPQIYTPLTAYGEDNQNESAQRVMTPLKSTMMISACSNNPGTVLVQNST
TPTVKALDMPPGDLSEIFFMNRKVMFDERKPTTTSWMTDTTDNPKTFTDCQKITITANKMAVPNEGSQMKMMTSDDQILYERQVITLKEGYMMTFSGNQTFTEDHKITSNNNYTLKWN
QMATLCEEQIMTLSDDQTFYRDQITFSGGQTLNGGQMETYIGDQTLYGGQMTFSGDQTLYGGQMNTLNDDQTLYGEQMNSRGDQIPYGGQMKTPSGDQTPCGGQMTTLKGGYTMTFIG
DQTFPGGQITTCSGVQTPYGSQMMMYKGDHMRTFTDDYNLYGDGGHMIPHQSSPQPYPGFLYFSSSHLTHGQYLEEQKCNIKTQKSKFQKKPDILKTYTCTYQDCGKSYTKPSHLRIH
ERKHTGEKPYKCNVKGCTWRFPRSDELNRHKRKHSGERPYLCTKCNRNFARSDHLKQHQRIHR
>panda FGENESH: 1 3 exon (s) 276 - 5969 583 aa, chain -
MGALGSALKGVAGQRRESARGAHPAGPYMKPGPAPSLAVLLRPAHDSTLSGPRAGAAYGESAPSNAGAKVRLPEPEDPITEDLVQLSSNSKENSTDSFLQTVEEIEEFLKDVFEIHQE
RASAAPEPQLGIPQTAHSEDSQHASAQKVMTPSRSTMMIPACSNIPGTILIQNSTLPTVKALEMPPEDLSETFPMNQKVTFFDERKPTTTSWMTDIIDNPETFAECQKVTVTANKIAI
PNEGSQMKMMTSDDQVLCERQVITLKEGHMMTVSGNQTFTEGHTMTSSGDHSLNWNQRTTLCEEQIKTPSGDQTLYGNQKNFSGGPMETYSGDQTLYGGQMTFSGDQTPYGGQMKMLC
DDQTLYGGQLTFRGDQILYGGQMKTLSDDQTLYGGQMTTLKGGYTVTFTGDQTFTGNQMTTYSGDQTPYGGQMMTLKGDHMTTFVDDHTLYGGHMMPHQSSSLPYPGFLYFSSSHLIH
GQSLEKQKCNIKTQSRQFQKNPDTLKNYTCTYQDCGKSYTKPSHLRIHERKHTGEKPYKCNVKGCPWKFPRSDELNRHKRKHSGERPYLCTKCNRNFARSDHLKQHQRIHK
>panda FGENESH: 1 2 exon (s) 1746 - 3861 507 aa, chain -
DPITEDLVQLSSNSKENSTDSFLQTVEEIEEFLKDVFEIHQERASAAPEPQLGIPQTAHSEDSQHASAQKVMTPSRSTMMIPACSNIPGTILIQNSTLPTVKALEMPPEDLSETFPMN
QKVTFFDERKPTTTSWMTDIIDNPETFAECQKVTVTANKIAIPNEGSQMKMMTSDDQVLCERQVITLKEGHMMTVSGNQTFTEGHTMTSSGDHSLNWNQRTTLCEEQIKTPSGDQTLY
GNQKNFSGGPMETYSGDQTLYGGQMTFSGDQTPYGGQMKMLCDDQTLYGGQLTFRGDQILYGGQMKTLSDDQTLYGGQMTTLKGGYTVTFTGDQTFTGNQMTTYSGDQTPYGGQMMTL
KGDHMTTFVDDHTLYGGHMMPHQSSSLPYPGFLYFSSSHLIHGQSLEKQKCNIKTQSRQFQKNPDTLKNYTCTYQDCGKSYTKPSHLRIHERKHTGEKPYKCNVKGCPWKFPRSDELN
RHKRKHSGERPYLCTKCNRNFARSDHLKQHQRIHK
>microbat /tmp/05_29_13-18:52:41.fasta|GENSCAN_predicted_peptide_1|631_aa
MTHTDHQGADALTETTDKLNETLKKSNETLEVVIKDQLEIKHTLTEIKDIMQTPNSRLEDCKNQVKDLKYEEAKSPNRKNKNKKESKNMKIVPCTEQPYASRPRLQTQWLLPALDEMM
DPTAEDLVQLSGNCQETHPLPQEIEEFVLEFSETHESRTPAAPEPQIYMPLTARGDESQHESIQNQVMAPLRPTMMASACSNIPGTVLDQNSITLPVNAFNKPPGELNETVSMDQKVT
SFDQRKPTATSWMIGVTDNPKTLFTDCQKTTITANNMTISKESSQMKTLSDEQTHYGGQMTFGGDQTGFGGQMTFGGDQTRYGDQMTFSGDQTRYGGQMTFGGDQTRYGGQMTFGGDQ
TRYGGQMTFSGDQTRYGGQMTFSGDQTRYGGQMTFGGEQTPYGGQMTFGGDQTRYGGQMTFGGDQTRYGGQMTFGGDQTRYGGQMTALKGGYPVTFIGNHTFTGGQVTTYSSDKTLYG
GPMGTLKGDQMRAFTDDHTLYGSHMMPCQSSSSPYPGFPYCSSSHLTQGQSLEEQQSTLKTQTCQFKKNLDILKPYICTYQDCQKSYSKKSCLQIHDRKHTGERPYKCNVKGCTWEFA
RSDELKRHNKKHSGERPYLCTLCDRRFARSDHLKQHQKVHQ
>megabat scaffold_742.7_prot length=500
MAGIVNKAGAEDLISAAGIIHGVEAKEGGALLQTDGGLQRRHLRGWSCWLLSSKAPQRLLEGIAADLLVPWNPPNHHLDPTAEDSVQLSGNSKETYTNSLLQTLEEIDEFLQDLSETH
EARTPAAPEPHLYIPLTAHGEDSQYEPIQNQAMTPLAPTMISACSNIPETVLVQNSTNPVKACDMPPGDLSETFSMNQRGISFGQRTPTATSWMTYVADSPNASFTDCQKITSTANMT
IHNEGSPMKTLSDDLTLCGGQMTSSGDQTHYGGLMTFGSDQSVDGGQMTFSGDQTHYGSLMTFGADQNVCGGQMTFSGDQTYYGGQTTFSGDQRTIHSGYQTLYEGQMMTLKGDHMTT
FTDHTLYGSHMMPCQSSSVPYPEFLCFSSSHLTQGQSLENQKSKLKSQRCRFQKNLDVSKPYTCTYQDCGKSYSKRSHLQIHERKHTGEKPYKCNWKGCTWEFARSDELNRHKRKHSG
ERPYLCTLCNKNFARSDHLKQHQRVHGL
>shrew gi|400654761|gb|AALT02127308.1| Sorex araneus contig127308, whole genome shotgun sequence FGENESH: 1 3 exon
(s) 18001 - 19828 432 aa, chain +
MEDKSQKRTTNIFHKNHAEFLQTCEAVDPAGPDPELCGPLTPQGPASTQEALDGQDILDQLLKELQIPRPVTLHAQGSPNEPCQNQANTLDTVPEGPQFSESLTPQGQGSPQLPTEDK
DSALNQLLEELMNTPSWEQNMSCSAQSNPTAAAHMTPDTDVLKTFTGDQTLQEGQPKTPGSEQSPCGYHVPYTGTQIINGIQLPFVGAQMLLANHIPFSGHQTPSGTQRSLTGQQTVF
GTPMSMSMNQSCSRNQVPFTGAQILLMNQVSFSGVHPINGNQMPHSGVPMIPVTGIPTVTFISNQTLTGSQHVTQSGIQRLYGIQMGPSLKSQKPKKRQRRKYHINADGLKPYACTFQ
GCGKQYAKPYQLRIHERVHTGDKPYICDVKGCPWKFARSDELSRHKKKHTGERPYRCPQCPMDFARADHLKQHARVHR
>elephant la_Klf18
MATSSGDQTFYKDQMTSLRGGHMTPSSGDQTYKGHMTTSSSDHTLCGDQLKTSSDFQTIRGGQMTTSNGDQTFCKDQMTSLNGGQVTTSNGDQIYKDQMTSLKGGQMTTSNDEQTYYK
DHMKASSSDHILCGDKMTTSSGYQNLCGGQMATSNDDQTFFKDQMTSLKEGYMTTCSGEQTYYKDQMTTSRSDHTLCADQMTTSSGYQTLCGGQMTNLKGDQVTTSRSDHTLCADQVT
TSSRYQTLCRGQMTTSNGDRTFSKDQMTSLKGGQVTTSNGGQTSSSDHTLYGDQMANTSGYQILSGGQITTSSGEQGYYKDQMTTSSSDHTLYGGQMMTSSGYQTFYGGQMTTFIGDK
DLYKDQMITLKEGQVTTSNGDRTSKNQVTTLKESQMTISSSDHTLYGDQITTSSGYQTLCGGQMTTSNGDQTFYKDQIFYKDQMTTLKESQMTTSSDFNLIQGQSLEEQKWKPKVQRR
QSQKNHDILKLYTCTYQDCGKSYLRRARLRIHECIHTGEKPYICNVKGCARKFSRSDGLYRHKKKHNGERPYVCTRCNKNFARSDHLKLHQKSHE
>manatee JH594621.438_prot length=700
MLLGSTLKGVANRRCQSGASAHLTKHFQSPGLASCSRVLVLLSLRQAVPNRVAESTSVESASPNAWAKTLGEIEFLQGLSEPCAEQANTPAAPEPQVYMPLTAHDNGSQHESTQNKVL
PSLRPTAMTSTHSDIPGTILTQNSTTPPVKALDMTFAGLSETFSMAQKVTSFDQIKPTAECQMTNITETTKTSFTDGQMTATTTSKMTNPNEGDQMMTLSVDQTLSGHQITISSGDQT
LSGDQLMTFDGDQALYGGQMTTLKGGQMMTSNGDQIYKDQKTTFKGDQLTFSSSDHTFYKHQMTSLKGGQMITFSGHHTLYWGQLMTSSSDQSIYGDQMTTFRGGQMMTSSGHHTFYG
GQLMTSSSDQNFYGYRMTTLKGGQLSTSSSDHTMTKSSGYQTLYRGQMTTFNGDQTFYEDQMTTSSSDHTLYGDQMTTSSGYQTLYRGQMTTFNGDQTFYKDQTTIGSNDHTLCGDQI
TTSSDSQTLCGSQMTTSNDDQTLYKDQMTTLKEGQMTTSNGEQTFYKDQMTNLKVGHMITYSSYKTIYGGLMTTLKRHHMTTSSGYYTLYRGYMMFHLVSSLPYWGFLYFSDFNLIQG
QSLEKQKWNPKAQRRQSQKNSDISKLYTCTYQGCGKSYSRHSRLRIHECIHTGEKPYICNVKGCTWKFSRLDGLNRHKRKHSGERPYLCTTCNKNFARSDHLKLHQKSHR
>elephant_shrew FGENESH: 1 2 exon (s) 1045 - 2526 353 aa, chain +
MSQTRSRLCSLQVPVLTSADSNNPGTLLTQNSTGDSIQAFEDLEETFPILTSSDQIKLTDFQMTNNIGIPNASFTASPKTNITANTMAILHQSNQMMTFNANQTPNGGQMTIFSDGQT
YYYKNQMEDSSDNQMMTFDGNQTLYGGQMPSSSDDEMMTFGGDHAFYGNQMATSSGNHTLSNDQTMVSNDQTMASSGYENLFGFQMTSLKVNEIMSYSRHQIQYGGPIVTPSGDHSLD
GNHERSQLVSSLSHQNLLYFSNVSVNQGQPMDQEQNWDPKIQKPNAVKPHMCPYQGCGKFFSKLSYFQKHQRIHTGMKVFVCNVKGCMQKFCHSDGLGRYKKIHSNERPYLCTVCNKG
FSRSAHLKQHFKTHKPATGREGRGGETEREREGEREKKSEKGGKKEYIQKNLHRSFTQDFKPINL
>cape_golden_mole FGENESH: 2 4 exon (s) 25236 - 37074 648 aa, chain -
MAKRRLSYFGHIVRRDQPLEKDIMFDKEEGQRKRGRPPTRWIGQVATSMGSSLATIVKLAQDWAVFHSAVHKVARTQPKVLVDSLGFSMYRTLEEIEFLQGLSEPCTEEAKPPAAPEQ
QSYMPLTIYDEGSPHESTQILVLPSLSSTMMTSAHSNILGTALSQNSTTGPIEDLELKTFGGVNEAFSMVTSFDQIKPMADCQMINTTDTPKISFIDNQETTITGSMMTIPNEGGQMM
NLSVNQTFNGGQITTSNDDQIFKDQMTALKRGQLMASINDQTFYGEQMTTFKGGQTMTSSGHHTLYGNQMMTSSSSQSFYRDHMMTSSGDQTFYKDQMTMFKGGQLMTSSSDQIFHGN

QLTTLKGNQLMTSINDQTFYGDQMTTSIGDQTLYGGQMTTSNGDQAFKDQMATFNGFQIMTSSGDQTSYGNYMTTLTGGQLATSNSDQTFCESLMTSLKRGQMTTSSEDHTLYGGQMT
TSSGYQTLLGCQMAMLKRSQMMTSSDHHTFYGGHMTSHLVSSLSCSESLYFSDAKLIQEQSLGKLKWDPKTQRHQFQKNPNILKPYACTYQDCEKSYSTHANLRKHEFIHTGKKPYIC
NVKGCTWKFSRSDGLKRHTKKHSGERPYLCTTCNRNFARSDHLKQHQKQHRQFLPINP
>/tmp/06_05_13-16:55:00.fasta|GENSCAN_predicted_peptide_1|243_aa
MTALQRSQLIASFNGQISCGHQMATTNDPRVLHGDQMTALQGSQLMTSSGHHSPYRGQLTTFHSDQSLYEGHTRTVNGSHVFYENLRTSFKRVQMETSNCDRAPYGGQMPTSRSYQTL
YGCRMTALKESYTLCGDQVTSHHASSSCSEFLCFSEVNLTQGQAPKEQKGNQKAQRRQFWTDPGVLKPYARTHQGCGKSSRSSSSKEGLHTHWGEVRHMQRKRMQGPPVKEHNAKPTA
AESTPCN
>rock_hyrax FGENESH: 1 2 exon (s) 6765 - 7359 153 aa, chain + | **zinc finger regions not detected in current genome with low coverage**
MTDTPKSSFTDDQMTAITASKMTVPNKDDFLRRPETAPKGGQMTTFRSDHTLYSGQVTPSSDYQTLYEAQMTTSSGDQTFYTDQVTTSSSDYTLYGDRMTTFIDYQTLCRGQMATSSD
DYTFCKDQMIVLSSDHTFYGDQMTMSSGYQILCGG
>armadillo JH568464.20_prot length=437
MSMDTFLQTLEDIDFHWDLSEPYKEQEKPWDVPEPQLYIPLTAPGEDMQHESTQNQVLPSLKLTMMTPVCCNIPGTVLTQTSTVPPVKVLDRPLGGLSGTCSMNQDLFDQIKPTASSQ
MTNVTDTPKASFTSDQKTTIIANKMTILNEHGQMMTLNSDQTLNEGQMTNQYRGHLKTLSDTQSICGGQMTALKGGQMMTSSGDQILYVEQMKTSSDDHDVYGGQTTISSGYHNLYGS
QMTMSSSYCSLYGGQMALPSDAQSLHGGQMTLFNGDHTLCRSQMMTLNGDQMTAFTDNHTLYGEGVIPHLSSSFPHPGFLYFSDSHLTQGVPEEKWNPKAHGCKRSCFQKKSDILKPY
TCKHQDCGKSFSKASYIQIHERVHSGEKPYSCDVEGCTWKFTRSDELSRHKRKHSGERPYPCTKCNRSFARSDHLKQHQKIHR
>sloth scaffold_49646.1_prot length=641
YPTTENLVQSSDNSKELSMDAFLQTLVETEFLRDPSKPHKEQAKPPDAPEPQLCISPTAHGEDSHHELTQNQVMPSSKPTVMTSICSEIPGMVLTQISAMTPAKVFDLPLGGLSGTCS
MDQDLFDQMKPTASSQMTNVTDTLKASFVTDQKTTISASKLTVFNEDGQTMTFNSDQTLNEGQKTNLCGSHLKTLSDAHSIFGGQMTTLKRGQIMTSSDDQILYGEQMKASSDYHNLH
GGQMTTSSGDQTLYGDQMKTSSGYHNLQGGQMTTSSGDQTLCGDQIKTSSGYHNLQGGQMMTSSVVYHNLHGGQMTTSSGDQTLKGNQIKTSSDYHNLQGGQMMTSSGDQTLYGDQMK
TSSGYHNLQGGQMTTSSGDQTLYGDQMKTSSVYHNFHGGQMSTSSGDHTLYGSQMKTSSDYHNLHGGQMSTSSGDQTLYGDQMKTSSGYQNLYGSQMTTSSCDQTLYGSQMTTLKGNQ
IMTSSGDQILYGSKMKTSSDCHSLYGGQMSTSSDDQTIYGNQMKTSSDYHSLYGGQMTFNCDHTLYMGQTMTLNGGQRTAFTDNHTVYGGHMFPHLSSSFPYPGFLYFSASHLIQGSP
EAQWNSEAHGCQRCCFQKKPDILKSYNCTFQGCGKSFSKPSYLQIHKRVHTGEKPYSCNVEGCTWKFSRTDELSRHKRRHSGERPYPCTKCNRSFARSDHLKQHERVHR

Section 2: predicted protein products of murine Zfp352/Zfp352l/Zfp353 genes

>mouse_Zfp352 gi|238859639|ref|NP_694742.2| zinc finger protein 352 [Mus musculus]
MEMAENTGASQPGPCNTQPSPVESSQLFSGQSSEMLTWKQTVMESASCSTQETTCTQNSTAYPGKAIGFHFGQPFENTSWNQQVVSAEQKISPAGLYHTQLNSAPQICLPPQKTTRVD
TRETFYNEDYWMRTLNSDKTLLTQNTAMCENQRVTFDNHQIALYGSHAGQDPNVGHAFASEHDWTSYNSHMTTRNGGHLTLYESQMTAPSCSQTLHPNQIITSFSEQNYFEDQQSNLA
VNNGFYGNQVILPNGYQAFYEPQMRANFDQTNDQMKSFSGQNVCKGQENLLSGECSLSGYQTSGYRCDQDLIVNPQVTSPIGGQPLSDFQTQTSSFDTALPGSKRTRPSAEDNLACPL
ETSPTSEETLYLGQMRTSVDQNFYSSQNGTPNIKESLDPQVTSFSLQAPYVGESSYPSCSPLIQKQPQKNSSASSLVQRQHPEMKLDFKTQSPVSQKDPSPLKLYFCTYKGCTKFYKR
AYHLKEHQKKHTDKRKYGCDEPGCTWSFFRLCDLNRHKEKHNGERFYACPLCSTNYSRLLYLKKHLEKKHAQAQPTTAT
>mouse_Zfp353 gi|18448208|gb|AAL69649.1| zinc finger protein 353 [Mus musculus]
MAENTGASKPRPCNTQPSPTESSQLVSGQSSEILTSKQTVMASASCSTQESTYTQNSTAYPAKAIGFHFGEPFENTSLNQQVVSAEQKISPAGFHHMQPNGTPKTFLPPQKTTRVDTR
ETFYNEDYWMRTLNSDKTLLIQNTAMCENQRVTFDNHQIALYGSHASQDPNVGHIFASEHDWTSYDSQMTTRNGGHLTLYGSQMTAPSYSQTLHPNQIITSFSEQNYFEDQQSNLAVN
NGFYEDQVILPNGYQAFYEPQMRANFDQTNDQMKSFSGQNVCKSQENLLSGECSLSGYQTSGYRCDQDLIVNPQVPSPIGGQPLSDFQIQTSSFDTTLPGSKRTRSSAEDNLACPLET
SPRSEETFYLGQMRTSVDQNVYPSQNGTPNIKESLDRKVISFSLQAPYVGEYSYSSHSPLIQKQPQKNSSASSLVQRQHPEMKLDFKTQSPVSQKNPSPLKLYFCTYEGCTKFYNRAY
HLKEHQKKHTCVRKYRCDEPGCTWSFFRLHDLNRHREKHSGERPYACPMCSTNYSRLVYLKKHLEKKHAHAQPTTAT
>mouse_Zfp352l FGENESH: 1 2 exon (s) 1624 - 3376 544 aa, chain -
LRNLKSSQAFCEVYMEMAEKENTGASQPGPCNTQPSPLEASQLVSGQSSEMPTWKQTVMVSASCSTQETACTQNSTVYMGKVIDFHFGEPEKIAVLDTRETLYSEESWMRTLSPDKNL
FIKTTVMCESQRIAFDIHQIALSGIYPDQEPIVDHLLAYVYDQTFQGELEITLRGDQISYYSHMTTRNGGHLTLYGSHMAAPSYSQALHPNQIITSFSEQNSFRDQQSKPAADNGFYG
DQVISPNGYQAFCELQMRANYDQTNDQMNSFSGQNVCKDQENLLIGDHSLSEYQTSGYRCDQDLIVNSQVTSPFGGQPLSDFQIPTSSFDTTLHGSKRTRSSAEDNLACPLETSPSSE
ETFYLGQMRTSIDQDVYPSQNGTLNIEESLDSQMTSLSYQVPYVGESSYPSSSPLIDPKSTSGKLFSFQSSPGTASRDEVRPQSPEPYVPEEPSSFECYSCTYANCQKSYKRAQHLEE
HMKKHTGEKPYACNKPGCTWKISCSKDLKRHKQKHSVVRPYPCPRCNKNFARLEYLKQHVRCHIEASFPTAT
>rat_Zfp352 gi|157819555|ref|NP_001102827.1| zinc finger protein 352 [Rattus norvegicus]
MDMAETPGASQTVPCNTQPSPVESSQLVSGQSSEMPTWKQTGMASSYFNPYETACTQNSTAHRGKATDSYFGEPSETASFIQQVVSAEQNKSPEGSLFMKLNSTATSCLPQPLDTRKT
LYNEGYWRRTLNSDKTLLIQNTDLCESQRITFGNHPVALSGSHAGQVPNVDHLDSVWDQTFGGDLEMPFSDDSYYSQMTTKYGGHLTLYESQMIAPSCNQVLDTDQFVTSFYEQNPFG
DQQRSPVTENKFYGDQMIVPNAYQAFYEPQVRDNSDQINFDSRMASFSGQNICSGQESIFQDEHSLSGFQTSGYRCDQDWIMNHQVISPIGGQTLYDSQKPTSTFNTTPHGSKRTTSS
AEDNLAWHPETSSSSEETLYMGQMRASVDQNVYASQNGTANTEESLEPEVTSLSNQAPDVGESSDSSSSPLIRIQSQGISSASGLDQGQHPQIRLDLKTQSPVSPEKPPTLKRYSCTY
QGCEKSYTKSHHLKDHMRKHTGEKPFVCDQIGCNWKFFRSIDLNRHKKKHSGERPYACPKCNKNYSRPYYLKQHQVSHIQALPTTAN
>rat_Zfp352l gi|293359360|ref|XP_233185.5| PREDICTED: uncharacterized protein LOC298232 [Rattus norvegicus]
MDVAETPGASQTVPCNTQPSPVESSQLVSGQSSEMPTWKKTVMASACCNPQETACTQNSTAHPGKASDFYFGEPSETASFIQQVVSAEQKSSPAGSCLMNLSSTTKSCLSHPLKTEVL
DTRKTLCNEGHWGRTLNSDKTLLIQITTMYESQWRTLGNHPVAPSGYHEGQAPNMDHLVDSVSNQTFIGDLEMEPSCNHVLYTDQFITSFSEQNPFGDQQRSPVADNRFYADQKILPN
GYQTFYEPQMSTNYQITCVCGQNVCRCQEILLSGEHLLSEYQTSGYRYDQDLIVNLQVTSPIGGEPLHDSQIPNSNFDTTSCGTKRTTSSEEDNLAWHPETSLSSEETFLDQMRTSVD
QNVYPSHNGTLKIEESLEPQVTSLSNQAPYVGESSDTSNSPLIQIQPQEISSASGLDQGQHPEIKLDLKTQSPVSLKNPHNSKRYCCTYQGCKKSYKKSQHLKDHMKKHTGVKPYMCN
KPGCDWKFFRLVDLNRHKQKHSGERPYPCPMCNKNYSRFYYLKQHLRSHIQASPTTAT
>rat_Zfp352lb FGENESH: 1 1 exon (s) 2130 - 3719 529 aa, chain +
MESSQLISAQSLEMPTWNQTTMTSVYSSTPKTTSTPNSTALPEHSDFHFGEPSEAASLHQHVVSTDQTQYPTFSHEMELNSASNSRLTHPQMIAVSDIRETAYNEDYWMKTLRSNWCE
SQKVVLGDNLVALCGSQADQDPNEDHLIASACDHYFRGNLEMPSSREQAYYTQMKSRNGGHLTLYETQMITANCSQNFLPNQIINSFSEQTPFGNQHGKAVADEMFSGDQMIASHGYQ
AFYEHQMTDSGDYINCDGEMTSWRNQNVNRGQVKLLGGDNTLYEHQASVYAADQDLCVDHQRSSPIGDQPLYDSQMSTLGFDTTLHVTNREKSNKENLSFHPRTISSSEEAFNWDQMK
TPFVQNVYPDQLGPLNVEESLDPQVTSLSNQTPYVVVSPCPSSSFLAQRQPLESSAASSLIQGQHPKMTSSLKTQNTHHLRPYLCAYQDCGKSYTKSHHLKDHMRKHTGEKPFVCNAP
ECEWKFTRLVDLLRHKNKHNRKRSYPCSMCNKSFSRLCYLRQHEKKKHSQSSPTNGT
