# Supplementary material for: A New Family of Predicted Krüppel-Like Factor Genes and Pseudogenes in Placental Mammals
Source: PLoS One. 2013 Nov 7;8(11):e81109. doi: 10.1371/journal.pone.0081109 (PMC3820594; doi:10.1371/journal.pone.0081109)

Figure S3. A phylogenetic tree of representative SP/KLF proteins with the human Wilms' tumor protein (human_WT1) as an out-group. This tree was generated by MrBayes. Each protein node is represented by the species name followed by the protein name.

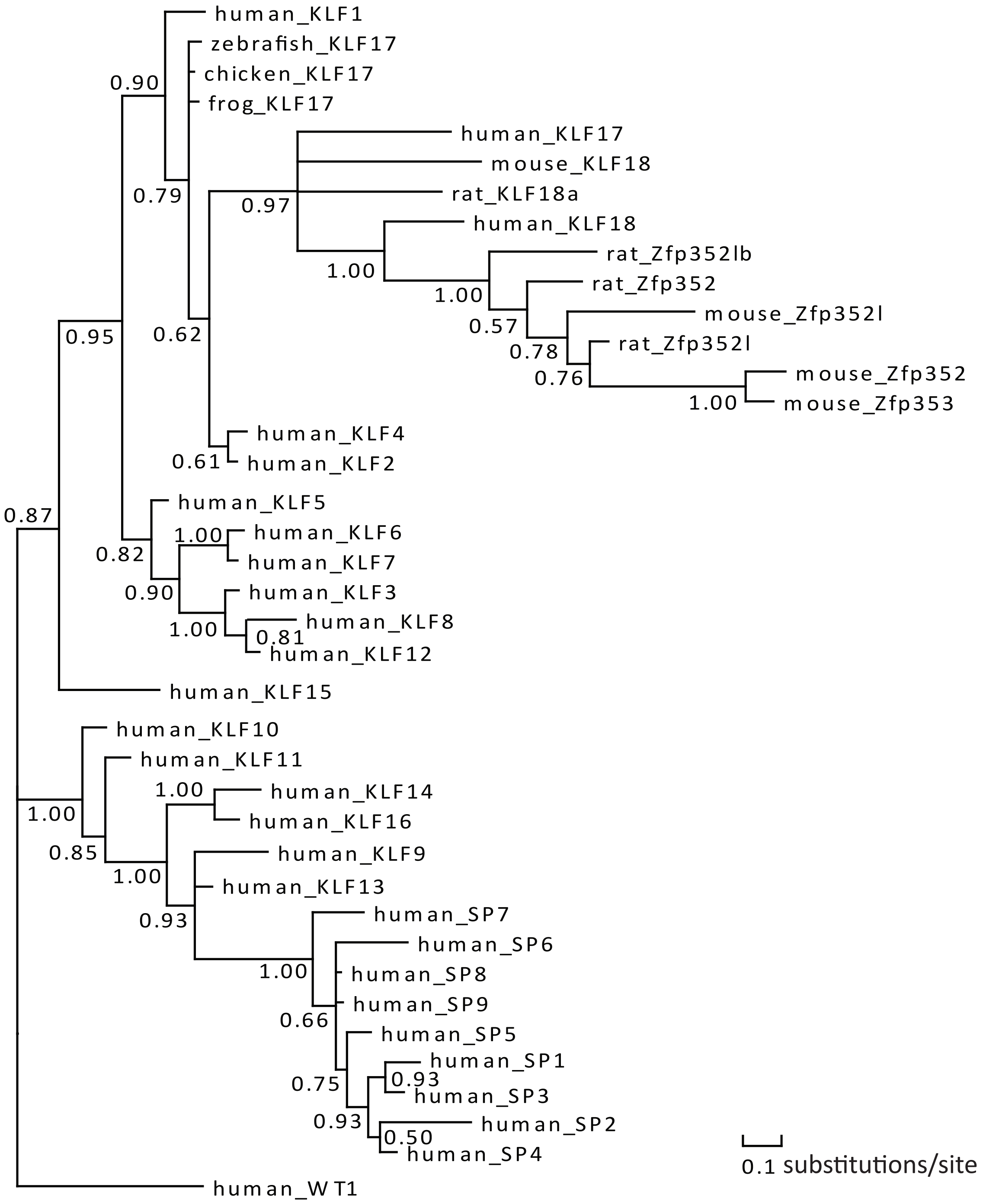

Supplement: Figure S3 — A phylogenetic tree of representative SP/KLF proteins with the human Wilms’ tumor protein (human_WT1) as an out-group. This tree was generated by MrBayes. Each protein node is denoted by its species name followed by the protein name. (PDF) [file pone.0081109.s004.pdf]
