# Supplementary material for: A New Family of Predicted Krüppel-Like Factor Genes and Pseudogenes in Placental Mammals
Source: PLoS One. 2013 Nov 7;8(11):e81109. doi: 10.1371/journal.pone.0081109 (PMC3820594; doi:10.1371/journal.pone.0081109)

Figure S4. A phylogenetic tree of SP/KLF proteins generated by MOLPHY. Each protein is denoted by its species name abbreviation followed by the protein name. Species name abbreviations are: bt, Bos taurus (cow); cf, Canis familaiaris (domestic dog); ch, Choloepus hoffmanni (two-toed sloth); cj, Callithrix jacchus (common marmoset); cp, Cavia porcellus (guinea pig); dn, Dasypus novemicinctus (nine-banded armadillo); do, Dipodomys ordii (kangaroo rat); dr, Danio rerio (zebrafish); ec, Equus caballus (horse); hs, Homo sapiens (human); la, Loxodonta Africana (African Savannah elephant); mm, Mus musculus (mouse); oc, Oryctolagus cuniculus (rabbit); rn, Rattus norvegicus (rat); sa, Sorex araneus (common shrew); tb, Tupaia belangeri (tree shrew); xt, Xenopus tropicalis (Western clawed frog).

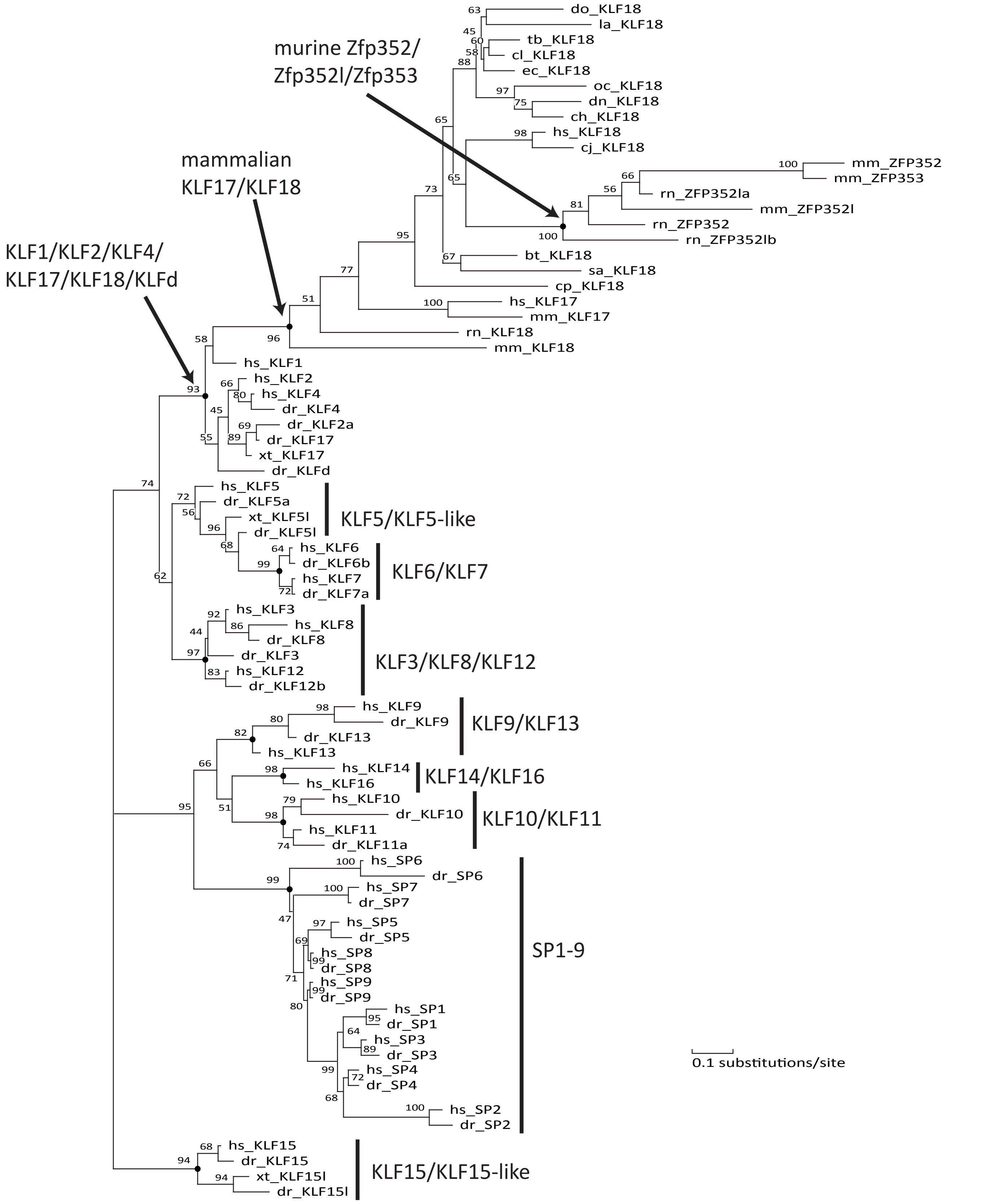

Supplement: Figure S4 — A phylogenetic tree of SP/KLF proteins generated by MOLPHY. Each protein is denoted by its species name abbreviation followed by the protein name. Species name abbreviations are: bt, Bos taurus (cow); cf, Canis familaiaris (domestic dog); ch, Choloepus hoffmanni (two-toed sloth); cj, Callithrix jacchus (common marmoset); cp, Cavia porcellus (guinea pig); dn, Dasypus novemicinctus (nine-banded armadillo); do, Dipodomys ordii (kangaroo rat); dr, Danio rerio (zebrafish); ec, Equus caballus (horse); hs, Homo sapiens (human); la, Loxodonta Africana (African Savannah elephant); mm, Mus musculus (mouse); oc, Oryctolagus cuniculus (rabbit); rn, Rattus norvegicus (rat); sa, Sorex araneus (common shrew); tb, Tupaia belangeri (tree shrew); xt, Xenopus tropicalis (Western clawed frog). (PDF) [file pone.0081109.s005.pdf]
